# Post-zygotic genomic changes in glutamate and dopamine pathway genes may explain discordance of monozygotic twins for schizophrenia

**DOI:** 10.1186/s40169-017-0174-1

**Published:** 2017-11-28

**Authors:** C. A. Castellani, M. G. Melka, J. L. Gui, A. J. Gallo, R. L. O’Reilly, S. M. Singh

**Affiliations:** 1 Department of Biology, The University of Western Ontario, London, ON N6A 5B7 Canada; 2 Department of Psychiatry, The University of Western Ontario, London, ON N6A 5B7 Canada; 3 Present Address: McKusick-Nathans Institute of Genetic Medicine, Johns Hopkins University School of Medicine, Baltimore, MD USA

**Keywords:** Schizophrenia, Whole genome sequencing, Monozygotic twins, Genome variation, Somatic mutation, Threshold model

## Abstract

**Background:**

Monozygotic twins are valuable in assessing the genetic vs environmental contribution to diseases. In the era of complete genome sequences, they allow identification of mutational mechanisms and specific genes and pathways that offer predisposition to the development of complex diseases including schizophrenia.

**Methods:**

We sequenced the complete genomes of two pairs of monozygotic twins discordant for schizophrenia (MZD), including one representing a family tetrad. The family specific complete sequences have allowed identification of post zygotic mutations between MZD genomes. It allows identification of affected genes including relevant network and pathways that may account for the diseased state in pair specific patient.

**Results:**

We found multiple twin specific sequence differences between co-twins that included small nucleotides [single nucleotide variants (SNV), small indels and block substitutions], copy number variations (CNVs) and structural variations. The genes affected by these changes belonged to a number of canonical pathways, the most prominent ones are implicated in schizophrenia and related disorders. Although these changes were found in both twins, they were more frequent in the affected twin in both pairs. Two specific pathway defects, glutamate receptor signaling and dopamine feedback in cAMP signaling pathways, were uniquely affected in the two patients representing two unrelated families.

**Conclusions:**

We have identified genome-wide post zygotic mutations in two MZD pairs affected with schizophrenia. It has allowed us to use the threshold model and propose the most likely cause of this disease in the two patients studied. The results support the proposition that each schizophrenia patient may be unique and heterogeneous somatic de novo events may contribute to schizophrenia threshold and discordance of the disease in monozygotic twins.

**Electronic supplementary material:**

The online version of this article (10.1186/s40169-017-0174-1) contains supplementary material, which is available to authorized users.

## Background

Monozygotic (MZ) twins originate from a single fertilized zygote and have been used to study the relative contribution of nature and nurture on a variety of phenotypes and disorders for well over a century. Schizophrenia, which is among the most devastating of the major mental health disorders, is thought to have both genetic and environmental causes [1]. Results show that although the frequency of schizophrenia is only 1% in the general population, its concordance in MZ twins approximates ~ 50% and not 100% [2]. Interestingly, recent results have shown that MZ twins may differ for de novo events, that include copy number variations (CNVs) [3, 4] and DNA methylation [5–8]. The timing, rate, extent and impact of such de novo events however, is difficult to ascertain. They may occur anytime during development as a normal aspect of development of an organ [9] and cell type [10]. Also, depending on the developmental stage at which they arise, will determine their presence in all or almost all cells of an individual or represent mosaicism. The random nature of this variation is expected to differ between MZ twins and contribute to twin differences. Although logical and attractive as a potential hypothesis for the cause of disease discordance in MZ twins, the confirmation of this hypothesis faces two challenges. The first is the identification of all or almost all differences between the genomes of discordant MZ twins and the second is establishing the significance of the observed genomic difference in the disease. Although the former is gradually becoming possible through increased resolution of genomic technologies, the latter remains challenging and will demand diligent efforts [11, 12].

The challenge of analysis and interpretation of complete genome sequences is attributed to a variety of factors [13]. First, the sequence coverage is not always 100% and second, the differences between MZ twins are not always easy to confirm due in part to expected mosaicism of unknown frequency and distribution [14], even though it is possible to differentiate between the pre-zygotic or post-zygotic origin of such twin differences using parental sequences. The next challenge is any involvement of observed differences in the development of schizophrenia. This of course is a much more complicated question. Schizophrenia is highly heterogeneous and affects ~ 1% of the world’s population with a heritability estimate of ~ 80% [1, 15]. Extensive research on this disorder has generated a large number of candidate genes. This includes a report involving 150,000 individuals with over 35,000 schizophrenic patients that has implicated over 108 genetic loci [16]. This considerable body of multifactorial and rather trustworthy data provides a standard for assessing the potential role of any new observation. We note that previously identified loci that are considered particularly trustworthy include *DRD2*, a common anti-psychotic target, as well as a number of glutamate receptors (*GRIA1, GRIN2A, GRM3*), members of the voltage gated calcium channels (*CACNA1C, CACNA1l* and *CACNB2*) and genes involved in synaptic plasticity [16]. As it stands, there are no common variants that account for a substantial portion of the liability to develop this disease [17]. It argues that the disease is highly heterogeneous and that most patients are genetically distinct. Also, any difference, particularly involving previously identified genes between monozygotic twins discordant for the disease, will have a high probability of being involved in the development of the disease. The results will be patient specific and may or may not apply to all or even most patients.

Finally, the results at hand allow us to appraise the threshold model for the development of schizophrenia [18]. It adds that inherited gene mutations [19–21] place an individual on a liability scale for the development of this disease. Additional contributors to this scale may include de novo mutations, environment factors and/or epigenetic [22] events, most occurring during ontogenic development [23]. The model predicts that the liability threshold may be met by inherited factors alone or require additional random genetic and epigenetic events including environmental events during development. We propose that this model can be tested using MZ twins discordant for schizophrenia with the assumption that inherited components will be shared between twins. Further, additional de novo mutations (and epimutations) will be acquired during ontogeny in the affected but not in unaffected twin. Given high heterogeneity of the disease, such results across MZD twin pairs may follow similar pattern but differ in the genes affected. Further, the genes affected may belong to a number of common pathways that appear to be invariably affected in this complex disease. Finally, the genes identified in this research are known to be involved in post-synaptic complexes [24] and synaptic strength [25], processes that are known to be defective in several but not all patients with schizophrenia.

## Methods

### Subjects

**Fig. 1:**
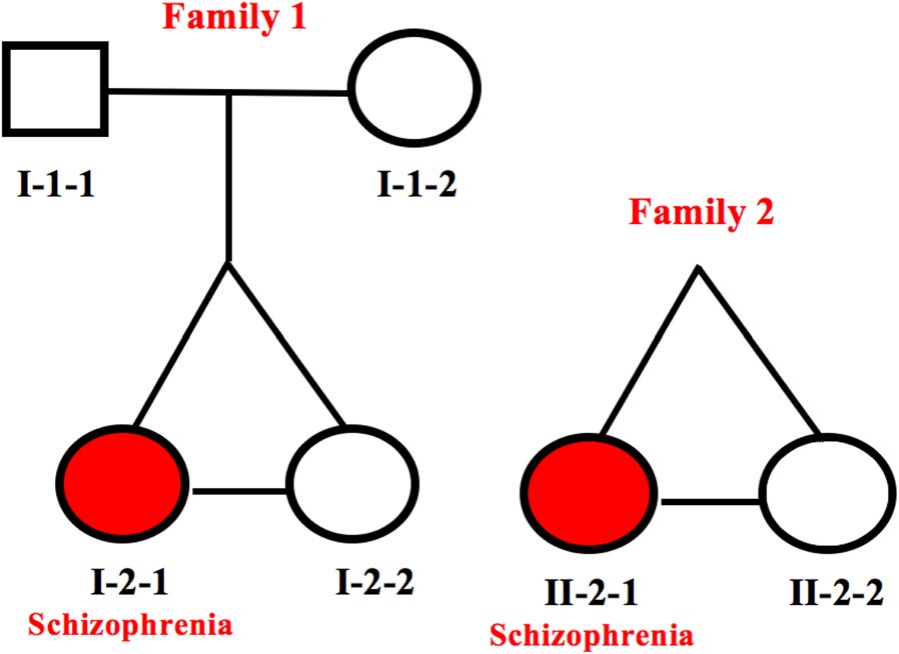
Members of the two discordant monozygotic twin pairs and one set of parents included in the study

**Fig. 2:**
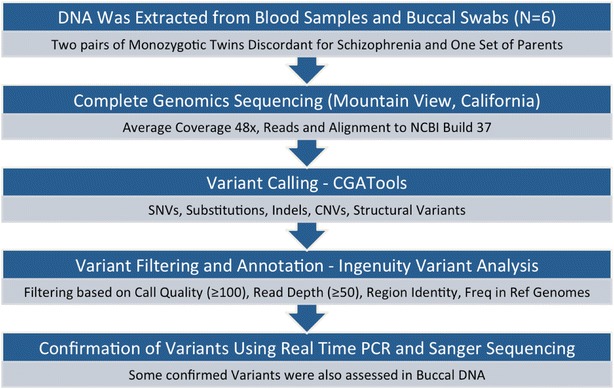
Flow chart of methods employed

This research received ethics approval from the Committee on Research Involving Human Subjects at The University of Western Ontario, London, Ontario, Canada. The methods were carried out in accordance with all approved guidelines and regulations. Two families with monozygotic twins discordant for schizophrenia were selected based on their long term clinical features assessed in-person by Richard O’Reilly (Psychiatrist), using the Structured Clinical Interview for DSM-IV (SCID I and SCID II) and a review of medical records from multiple decades [26, 27]. All participants provided written informed consent. A second senior psychiatrist independently reviewed videotapes of the structured interviews of the twins and confirmed the diagnoses. It has assured a reliable diagnosis and long-term discordance of the two MZ twin pairs used in this study. All six subjects (Fig. 1) provided blood and cheek swab samples that were used to isolate genomic DNA using the PerfectPure DNA blood kit (Blood samples), and the QIAGEN DNA Micro Kit (Buccal samples), following the manufacturer’s protocols. The experimental workflow of the analysis is summarized in Fig. 2.

The twins from family 1 are Afro-American females and contributed DNA samples at age 53. The affected twin (I-2-1) was diagnosed with schizophrenia at age 22. She was admitted to a psychiatric unit on at least 12 occasions and experienced paranoid delusions, auditory hallucinations and occasionally euphoria during her acute episodes of illness. This twin had significant functional decline and lived with her parents throughout her adult life. The diagnosis of schizophrenia was confirmed by the study psychiatrist. I-2-2 did not experience any significant symptoms of mental illness until she developed a brief episode of depression at age 48, which was treated with an antidepressant. Four years later this twin developed an acute episode of mania, which required hospitalization and she was placed on the mood stabilizer divalproex sodium. She was diagnosed as having a bipolar disorder by the study psychiatrist. This twin pair has been previously described in detail [28]. At the time of sample collection, the twins were discordant for schizophrenia for 31 years. The father (I-1-1) of the twins was aged 82 at assessment and had a mild obsessive–compulsive personality disorder but no other psychiatric problems, while the mother (I-1-2) was aged 74 and had never had any psychiatric problems.

At the time of sample collection, the twins from family 2 (Fig. 1) were 43 years of age. The twins are Caucasian females. Twin II-2-1 became ill at age 27. She experienced paranoid delusions and hallucinations, which usually occurred in the context of a euphoric or depressed mood. She lived with her twin sister and had worked intermittently. She was diagnosed with a schizoaffective disorder. The twin sister (II-2-2) sustained skeletal injuries in a motor vehicle accident at age 18 years. A boyfriend died close to the time of this accident and II-2-2 became depressed for a short period around this time. She has had no further episodes of depression or other emotional or psychiatric problems. The twins were considered discordant for 16 years at the time of sample collection.

### Genome sequencing

The genome sequence of the six subjects (Fig. 1) was generated at Complete Genomics Inc. (Mountain View, CA) in the form of ~ 2 billion overlapping 70-base nucleotide sequences. They allowed reconstruction of six individual genomes. Duplicates were removed prior to variant calling. The sequences met the criteria of high accuracy (99.999%) and were considered suitable for identification of rare variants including somatic mutations as described by Drmanac et al. [29]. These variants included single nucleotide variants (SNVs), indels and block substitutions as well as larger variants classified as CNVs and SVs that were called in comparison to reference sequence (NCBI Build 37). CNVs were called based on a read depth or depth-of-coverage algorithm provided by Complete Genomics. Sequence coverage was averaged and then GC bias was corrected for over a fixed window (2 kb) and normalized relative to a set of standard (CG 45 genome reference) genomes sequenced by Complete Genomics. A Hidden Markov Model was used to determine the integer copy number state (0–10). Structural variations were detected by identifying discordant mate pair mappings found during the assembly process. Mate pair mappings where each arm maps to the reference genome but with either an unexpected length between them or an anomalous orientation are subjected to local de novo assembly to refine junction breakpoints at single base pair resolution. Complete genomics then generated a high confidence junctions file that reports junctions that are most likely to be accurate. CGAtools junctions2events was then used to convert high confidence junctions to possible SV events using repeat masker 37 data, hg19 reference file and a refseq gene information file. The junctions2events command in CGAtools identifies likely deletion, inversion and translocation events from the list of high confidence junctions delivered by Complete Genomics. SVs were further refined into five categories: deletions, tandem duplications, distal duplications, inter-chromosomal events and inversions. To call small variants in our dataset, Master Variation Files (MasterVar) were generated using CGAtools and hg19 as reference. VCF files were then generated from each MasterVar file using CGAtools command mkvcf. VCF files generated this way were directly inputted into ingenuity variant analysis (Ingenuity Systems, Redwood City, CA). Given the potential for false positives identified in genome sequencing, a stringent read depth of 50 and a call quality of 100 (calculated by complete genomics and based on a phred scale) were chosen as parameters for initial variant filtering. In the analysis of family 1, variants that were present in the affected twin and not present in the unaffected twin or their mother or father were assessed as de novo. Also, in the twin analyses of family 2, without parents, variants that were present in the affected twin and not present in the unaffected twin were labeled as *provisional de novo*. Variants were annotated with overlapping genes, cytoband, gene region (Exonic, Intronic, Promoter, 5′UTR, 3′UTR, Splice Site), translational impact (if applicable), SIFT function prediction (if applicable), SIFT score (if applicable), dbSNP ID (if applicable) and frequency in the 1000 Genomes as well as frequency in the Complete Genomics Public Genome dataset. The biological context filter in ingenuity variant analysis was applied to downstream data with “Schizophrenia, Neurological Disease and diseases consistent with those two phenotypes” as the filtering criteria. Genes had to have a direct connection to these phenotypes (no hops allowed).

In the case of CNVs and SVs a 50% reciprocal overlap rule [30–32] was applied to determine if two variants were the same or different between twins/parents. All CNVs/SVs had to meet the ≥ 50% rule when compared to the other CNV/SV, otherwise, the two were considered unique. Pairwise comparisons were performed for all CNVs and SVs. To increase the efficiency of this, HD-CNV was utilized [33]. Comma delimited files were prepared using a custom python script and the 50% reciprocal overlap rule was applied to determine the CNVs and SVs that were unique to an individual. Interchromosomal and inversion comparisons were analyzed manually due to the limitations of HD-CNV. Inter-chromosomal events were considered the same if the origin and the destination chromosome numbers matched and the junction positions were less than 500 bp apart. Inversions had to share the same direction and 50% or more identity to be classified as shared.

### Confirmation of variants

Quantitative PCR using TaqMan^®^ Assays in an Applied Biosystem StepOne were performed on selected CNVs [4] and SVs [6]. CNVs and SVs that were confirmed via Real Time PCR were then assessed in the Buccal DNA from the same individuals to identify if the variant arose before or after the differentiation of the germ layers. RNAseP was used as the reference gene. Predesigned TaqMan assays were ordered from Life Technologies for regions of interest. CopyCaller 2.0 was used to generate the predicted copy numbers. Circos [34] was used for visualization of post-filtering variation data. Unique variants were separated into files to generate relevant tracks in the genome diagram. Structural variants that involved more than one chromosome were visualized using “links” in Circos [34].

Sanger sequencing was used to assess small variants of interest in our dataset [28]. Regions were sequenced at the DNA sequencing center at The London Regional Genomics Centre. Variants that appeared mosaic in nature were further assessed using PASA (PCR amplification of specific alleles).

### Pathway analysis

Any unique variant that overlapped a gene was identified and the gene lists were used in Core pathway analysis (Ingenuity Systems, Redwood City, CA) to identify pathways and networks that were overrepresented in the filtered dataset. Only the genes that were identified to overlap high confidence somatic de novo (family 1) or *provisional de novo* (family 2) variations were included in the pathway analysis. Variants that were found to be unique to unaffected twins were also assessed in a separate pathway analysis. The top 20 canonical pathways found to be overrepresented in each individual were then compared between all members in the study; pathways that were found in affected and unaffected twins were labeled as GP. They were viewed as “shared genetic predisposition” between the two members of the twin pair. Pathways that were unique to affected twins only were labeled as GPD. They were viewed as additional “genetic predisposition that led to disease” in the affected and not the unaffected member of the twin pair.

## Results

### Defining genetic variation including de novo events

The complete genome sequences of two MZD twin pairs and one set of parents (Fig. 1) were generated with a high call quality above 100 (Additional file 1: Figure S2) and an average read depth of 47–50 fold coverage (Additional file 1: Figure S3) following our workflow (Fig. 2). They represented over 99% of the reference sequence for each genome (Table 1). We compared individual genome sequences with the Genome Reference Consortium Human Genome Build 37 using Complete Genomics Analysis tools (CGAtools) for identification of individual specific variants and their genomic location at single base pair resolution. The results show that each genome harbors 3.3 to 3.9 million single nucleotide variants (SNVs), 370–430 thousand indels (small insertions and deletions), 71–80 thousand block substitutions, 1 thousand structural variations, and 150 copy number variations as well as a transition/transversion ratio of 2.1. The exception to this pattern was the father in family 1 who carried 592 copy number variations and 1110 structural variations, that may be associated with a diagnosis of chronic leukemia unrelated to this study [35]. We further grouped individual variations as small sequence changes, (SSCs—SNVs, indels, block substitutions), copy number variants (CNVs) or structural variants (SVs). An example to structural variation observed in family 1 involving inter-chromosomal translocation is depicted in the Circos plot (Additional file 1: Figure S1).

**Table 1:** Identity of sequenced genomes

Sample ID	Family	Gender	Age at assessment	Age of onset	Disease status	% of genome called	Normalized average coverage
I-1-1	Family 1	Male	80	N/A	Unaffected	99.037	49.55
I-1-2	Family 1	Female	76	N/A	Unaffected	99.042	47.27
I-2-1	Family 1	Female	43	27	Affected	99.042	48.07
I-2-2	Family 1	Female	43	N/A	Unaffected	99.042	47.41
II-2-1	Family 2	Female	53	22	Affected	99.039	49.71
II-2-2	Family 2	Female	53	N/A	Unaffected	99.037	50.25

### Distribution of variants across twins

**Fig. 3:**
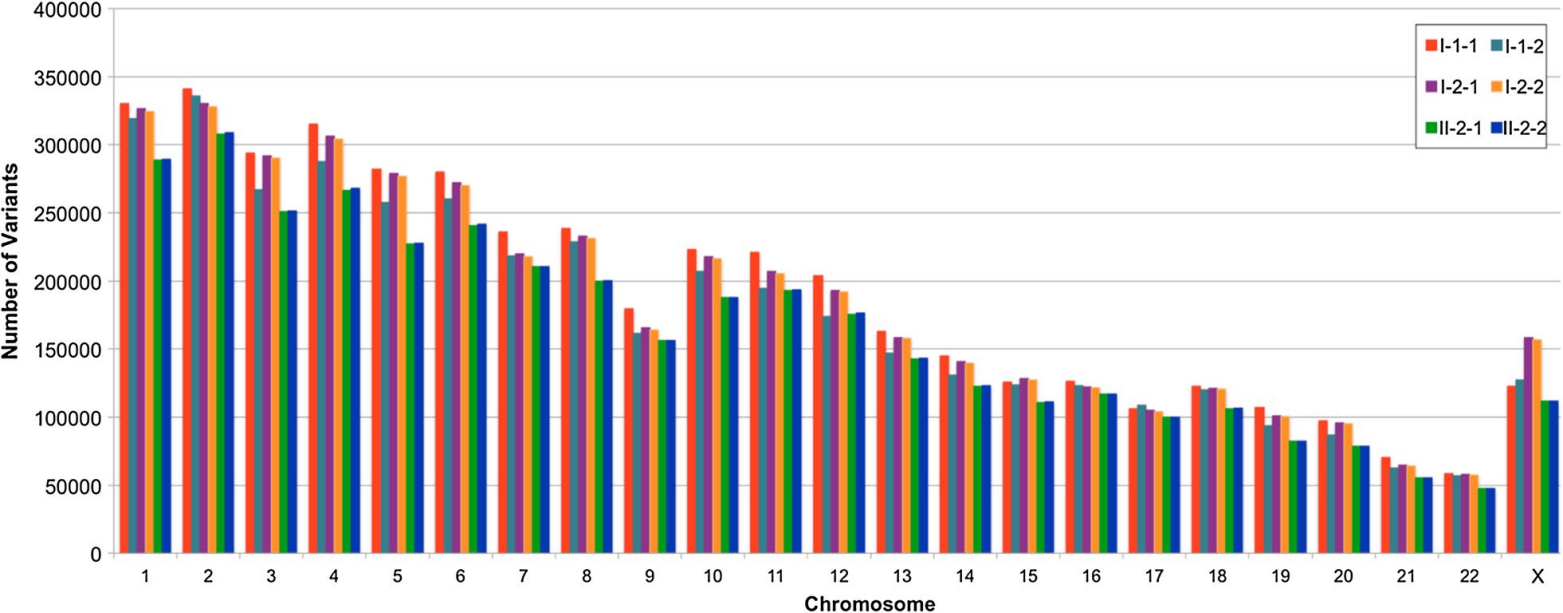
Chromosomal distribution of small sequence variations (SNVs, Indels, block substitutions) in all six samples

The frequency of variants across chromosomes that is related to their size is different between parents at each chromosome but rare between the monozygotic twins (Fig. 3). In family 1, some sequence differences between twins were viewed as inherited (present in at least one parent) and others were considered as post zygotic de novo given that they were not present in any of the two parents (Table 2). The de novo variants were further assessed by their presence in exons (Tables 3 and 4). In family 1, two unique CNVs were identified as inherited and five were considered de novo in the affected twin (Table 5). In contrast, in the unaffected twin, five CNVs were identified as inherited and four were labelled as de novo. The CNVs unique to the affected member of twin pair 1 and 2 are given in Tables 6 and 7 respectively. Interestingly, the structural variations followed a pattern similar to small sequence variations (Table 8). Most of these variants are shared between twins, the unshared variants are more common in the affected as compared to their unaffected counterpart and the post-zygotic de novo events are not different between MZD twin in family 1. In the absence of parental sequences for family 2, it was not possible to consider the familial vs somatic de novo nature of the twin differences. For the purpose of this discussion variations that were unique to the affected twin in twin pair 2 were considered as “*provisional de novo*”. We conclude that most human variations are historical, yet rare variations may be acquired through de novo events during genetic transmission as well as extensive mitosis during development and aging. As expected, the post zygotic de novo variants are often reflected as different degrees of mosaicism, that are not always easy to independently confirm using standard technologies.

**Table 2:** Shared and de novo small sequence variations (SNVs, substitutions, Indels) as compared to NCBI Build 37

Sample	Total sequence variations before confidence filters applied	High confidence variants unshared with co-twin	Unshared with co-twin	
			Inherited (present in parent)	De novo (not present in parent)
Family 1 affected (I-2-1)	4,295,920	11,577	7302	4275
Family 1 unaffected (I-2-2)	4,265,089	9345	5776	3569
Family 2 affected (II-2-1)	3,780,127	10,725	N/A	N/A
Family 2 unaffected (II-2-2)	3,789,298	10,351	N/A	N/A

**Table 3:** Exonic de novo variants in the affected twin in family 1 (I-2-1)

Chr	Position	Ref	Sample	Type	Cytoband	Gene	Translation impact	dbSNP ID	1000 genomes	CG public genomes
1	117142868	CA	TG	Sub	1p13.1	**IGSF3**	In-frame			
4	85996		C	Ins	4p16.3	**ZNF595**	***Frame shift***			
4	86004	A	G	SNV	4p16.3	**ZNF595**	***Missense***	112290390		
4	86022	CAG	GAC	Sub	4p16.3	**ZNF595**	In-frame	386670355		
4	86043	T	C	SNV	4p16.3	**ZNF595**	Synonymous	377364445		
7	100549935	T	G	SNV	7q22.1	**MUC3A**	***Missense***	73714229		
7	100549942	T	C	SNV	7q22.1	**MUC3A**	***Missense***	73714230		
7	100552452	T	C	SNV	7q22.1	**MUC3A**	Synonymous	112050489		
11	48367133	C	A	SNV	11p11.2	**OR4C45**	***Missense***	79453749		
12	40875389	CCATCAGCTGGAGTGACAGTGACATCCGGA		Del	12q12	**MUC19**	In-frame			
12	40880542	C	T	SNV	12q12	MUC19	Missense	370502411		14.81
12	40880545	C	G	SNV	12q12	MUC19	Missense	199768257		16.66
12	122359397		GAGGAGGAGGAGAAA	Ins	12q24.31	WDR66	In-frame	142042908		67.59
13	25671607	GA	AG	Sub	13q12.13	**PABPC3**	In-frame	386769143		
13	46170723	GATACTCTTCCTCCTCCA		Del	13q14.13	**ERICH6B**	In-frame			
17	74288565	TGA		Del	17q25.1	QRICH2	In-frame	35035566	46.96	20.37
22	29885594	A	T	SNV	22q12.2	**NEFH**	Synonymous	79235463		
22	29885599	AGGAAG		Del	22q12.2	**NEFH**	In-frame	149571560		

Gene variants in bold are not known to be polymorphic and considered novel and potential pathogenic impact is marked in bolditalic

**Table 4:** Exonic de novo variants in the affected twin in family 2 (II-2-1)

Chr	Position	Ref	Sample	Type	Cytoband	Gene	Translation impact	dbSNP ID	1000 genomes	CG public genomes
1	1650787	T	C	SNV	1p36.33	CDK11B; CDK11A	Missense	1137003		28.7
1	1650797	A	G	SNV	1p36.33	CDK11B; CDK11A	Missense	1059830		31.48
1	1650801	T	C	SNV	1p36.33	CDK11B; CDK11A	Synonymous	1137004		31.48
1	13183225	T	C	SNV	1p36.21	**HNRNPCL1/HNRNPCL2**	Synonymous	28634306		
1	13183228	C	T	SNV	1p36.21	**HNRNPCL1/HNRNPCL2**	Synonymous	144054379		
1	13183237	CT	TC	Sub	1p36.21	**HNRNPCL1/HNRNPCL2**	In-frame			
1	13183243	T	C	SNV	1p36.21	**HNRNPCL1/HNRNPCL2**	Synonymous	138897759		
1	13183248	TC	AA	Sub	1p36.21	**HNRNPCL1/HNRNPCL2**	In-frame			
1	17266536	G	C	SNV	1p36.13	CROCC	Missense	9435714		10.18
1	62675619	C	T	SNV	1p31.3	L1TD1	Synonymous	4625314	29.85	33.33
1	109007867	G	A	SNV	1p13.3	NBPF4/NBPF6	Missense			26.85
1	109007877	T	G	SNV	1p13.3	NBPF4/NBPF6	Synonymous			26.85
1	109737063	C	T	SNV	1p13.3	KIAA1324	Synonymous	386565601	71.37	68.51
1	109737090	G	A	SNV	1p13.3	KIAA1324	Synonymous	386565600	71.53	67.59
1	111957583	A	G	SNV	1p13.2	OVGP1	Missense	1126656		3.7
1	111957592	A	G	SNV	1p13.2	OVGP1	Missense	56294468	22.32	3.7
1	120539742	G	A	SNV	1p12	**NOTCH2**	***Missense***	2258139		
1	120548025	G	A	SNV	1p12	**NOTCH2**	Synonymous	140551270		
1	120548055	T	C	SNV	1p12	**NOTCH2**	Synonymous	199592384		
1	144873963	T		Del	1q21.1	PDE4DIP	Frame shift		28.91	32.4
1	144922523	C	T	SNV	1q21.1	PDE4DIP	Missense	2455994		28.7
1	145103928	G	A	SNV	1q21.1	SEC22B	Synonymous	2596251	34.01	35.18
1	201178819	G	A	SNV	1q32.1	IGFN1	Missense	72468019		11.11
1	206566904	T	C	SNV	1q32.1	SRGAP2B; SRGAP2D; SRGAP2; SRGAP2C	Synonymous	2919105	49.44	
1	248813827	T	C	SNV	1q44	**OR2T27**	***Missense***	1782241		
2	132238043	A	C	SNV	2q21.1	TUBA3C/TUBA3D	Synonymous	74625243		14.81
3	75786499	CA	TG	Sub	3p12.3	ZNF717	In-frame			0.92
3	75786515	GG	AT	Sub	3p12.3	ZNF717	In-frame		0.92	
3	75787265	C	G	SNV	3p12.3	**ZNF717**	Synonymous	144538707		
3	75787269	G	T	SNV	3p12.3	**ZNF717**	***Missense***			
3	75788023	C	T	SNV	3p12.3	ZNF717	Missense	76889571		50
3	100170634	T	C	SNV	3q12.2	LNP1	Synonymous	9848109		24.07
3	195510827	C	T	SNV	3q29	MUC4	Missense	413807	3.53	24.07
3	195511780	G	A	SNV	3q29	**MUC4**	***Missense***	391928		
5	140725160		A	Insertion	5q31.3	**PCDHGA3; PCDHGA2; PCDHGA1**	***Frame shift***	372306793		
6	26406255	G	A	SNV	6p22.2	BTN3A1	Synonymous	3857550		41.66
6	33037579	AT	TG	Sub	6p21.32	HLA-DPA1	In-frame	386699858		37.96
6	33052986	T	C	SNV	6p21.32	HLA-DPB1	Synonymous		46.37	37.03
7	100550039		CTC	Ins	7q22.1	MUC3A	In-frame			
7	142460313	T	C	SNV	7q34	PRSS1	Synonymous	6666	39.66	24.07
7	142460335	A	G	SNV	7q34	PRSS1	Missense	201550522	0.16	
8	10467652	GC	CT	Sub	8p23.1	**RP1L1**	In-frame	386722180		
8	103573011	TGCAACCCCTGCAGCCCCTGCAACCCG		Del	8q22.3	ODF1	In-frame		31.29	23.14
11	1016776	C	T	SNV	11p15.5	MUC6	Missense	33988517		38.88
11	1017110	CGGT	TGCC	Sub	11p15.5	MUC6	In-frame			32.4
11	1018069	G	A	SNV	11p15.5	**MUC6**	***Missense***	10736904		
11	1018090	G		Del	11p15.5	**MUC6**	***Frame shift***			
11	1018095	G	TA	Sub	11p15.5	**MUC6**	***Frame shift***			
11	1092684	C	T	SNV	11p15.5	**MUC2**	Synonymous	201269049		
11	5270686	G	A	SNV	11p15.4	HBG1	Missense	1061234		75.92
11	18290866	T	C	SNV	11p15.1	**SAA1**	Synonymous	1136745		
11	48346916	G	C	SNV	11p11.2	**OR4C3**	***Missense***	77069283		
11	48346924	T	C	SNV	11p11.2	**OR4C3**	Synonymous	72911454		
11	48346932	G	A	SNV	11p11.2	OR4C3	Missense	80285195	0.28	
11	48347306	G	T	SNV	11p11.2	OR4C3	Missense	73465911		33.33
11	48367052		AG	Ins	11p11.2	**OR4C45**	***Frame shift***			
11	48367073	A	C	SNV	11p11.2	OR4C45	Missense	7941588	79.55	75
11	56143357	G	A	SNV	11q12.1	OR8U1; OR8U8	Synonymous	76949582		36.11
11	56143963	AATCTATAGC	GATTTACAGT	Sub	11q12.1	**OR8U1; OR8U8**	Synonymous			
11	56468020	T	C	SNV	11q12.1	OR9G1; OR9G9	Missense	532637	41.21	56.48
11	57982726	G	T	SNV	11q12.1	OR1S1	Synonymous	1993089	52.32	52.77
11	57982763	A	G	SNV	11q12.1	OR1S1	Missense	61763008		50.92
11	89703619	G	A	SNV	11q14.3	TRIM64/TRIM64B	Missense	79824618		77.77
11	89819403	G	T	SNV	11q14.3	**UBTFL1**	***Missense***			
12	11214857	T	C	SNV	12p13.2	**TAS2R46; PRH1**	***Missense***	201891491		
12	11214870	A	T	SNV	12p13.2	**TAS2R46; PRH1**	Synonymous	200226376		
12	11244721	GAA	CAC	Sub	12p13.2	TAS2R43; PRH1	In-frame			19.44
12	11244730	AA	GG	Sub	12p13.2	TAS2R43; PRH1	In-frame			18.51
12	49522578	T	C	SNV	12q13.12	TUBA1B	Synonymous	1057725	54.49	
12	49522605	C	T	SNV	12q13.12	TUBA1B	Synonymous	1057548	45.33	
12	52843610	C	A	SNV	12q13.13	**KRT6B**	***Missense***			
13	46170724	ATACTCTTCCTCCTCCAG		Del	13q14.13	**ERICH6B**	In-frame			
13	53216666	G	A	SNV	13q14.3	HNRNPA1L2	Synonymous	113869751	1.08	1.85
13	103399222	G	T	SNV	13q33.1	CCDC168	Stop gain			25
18	9887394	C	T	SNV	18p11.22	TXNDC2	Synonymous	2240910	51.84	
19	4511197	G	A	SNV	19p13.3	**PLIN4**	Synonymous	199625614		
19	4511200	C	T	SNV	19p13.3	PLIN4	Synonymous	200718202	0.14	
19	55286854	A	G	SNV	19q13.42	KIR2DL1/KIR2DL3	Missense	666590	0.08	
19	55286864	A	C	SNV	19q13.42	KIR2DL1/KIR2DL3	Synonymous	77397437	16.63	
21	10942923	G	A	SNV	21p11.1	TPTE	Missense	76723236		7.4
22	38120180	CTC		Del	22q13.1	**TRIOBP**	In-frame			
22	39387558	C	T	SNV	22q13.1	**APOBEC3A_B; APOBEC3B**	Synonymous	1065184		

Gene variants in bold are not known to be polymorphic and considered de novo and potential pathogenic impact is marked in bolditalic

**Table 5:** Shared and unshared copy number variants (CNVs) as compared to NCBI Build 37

Sample	Total CNVs	Shared with co-twin	Unshared with co-twin	Shared with co-twin		Unshared with co-twin	
				Inherited	De novo	Inherited	De novo
Family 1 affected (I-2-1)	152	145	7	131/145	14/145	2/7	5/7
Family 1 unaffected (I-2-2)	154	145	9	131/145	14/145	5/9	4/9
Family 2 affected (II-2-1)	156	151	5	N/A	N/A	N/A	N/A
Family 2 unaffected (II-2-2)	157	151	6	N/A	N/A	N/A	N/A

**Table 6:** Copy number variants unique to the affected member of family 1 (I-2-1)

Chr	Start	End	Cytoband	Size (bp)	CNV type	Identity	Overlapping genes
5	119380128	119382128	5q23.1	2000	Del	De novo	None
9	6700000	6710000	9p24.1	10,000	Del	De novo	None
10	26998675	27002675	10p12.1	4000	Del	De novo	PDSS1
14	20314000	20328000	14q11.2	14,000	Amp	Inherited	None
15	22422114	22492114	15q11.2	70,000	Amp	De novo	LOC642131
16	34467150	34515150	16p11.2	48,000	Amp	De novo	None
16	55841801	55855801	16q12.2	14,000	Amp	Inherited	CES1

The parental sequences have been used to establish the de novo [absent in parent(s)] or *inherited* [also present in parent(s)] nature of each CNV. Shared identity is based on a 50% reciprocal overlap rule

**Table 7:** Copy number variants unique to the affected member of family 2 (II-2-1) as compared to unaffected MZ twin

Chr	Start	End	Cytoband	Size (bp)	CNV Type	Overlapping genes
5	17612657	17620657	5p15.1	8000	Del	None
5	46244657	46246657	5p11	2000	Amp	None
5	135114128	135120128	5q31.1	6000	Del	None
12	114521470	114529470	12q24.21	8000	Amp	None
14	106926000	106930000	14q32.33	4000	Amp	None

Shared identity is based on a 50% reciprocal overlap rule

**Table 8:** Shared and unshared structural variation (SV) as compared to NCBI Build 37

Sample	Total SVs	Shared with co-twin	Unshared with co-twin	Shared with co-twin		Unshared with co-twin	
				Inherited	De novo	Inherited	De novo
Family 1 affected (I-2-1)	919	781	138	750	31	97	41
Family 1 unaffected (I-2-2)	893	781	112	750	31	66	46
Family 2 affected (II-2-1)	996	855	141	N/A	N/A	N/A	N/A
Family 2 unaffected (II-2-2)	977	855	122	N/A	N/A	N/A	N/A

Structural variants fell under five categories: deletions, tandem duplications, distal duplications, inter-chromosomal variations and inversions. There were no inversion differences found between twins in either family. They were not included in subsequent analyses

#### De novo mutational features in twins affected with schizophrenia: SSCs

**Fig. 4:**
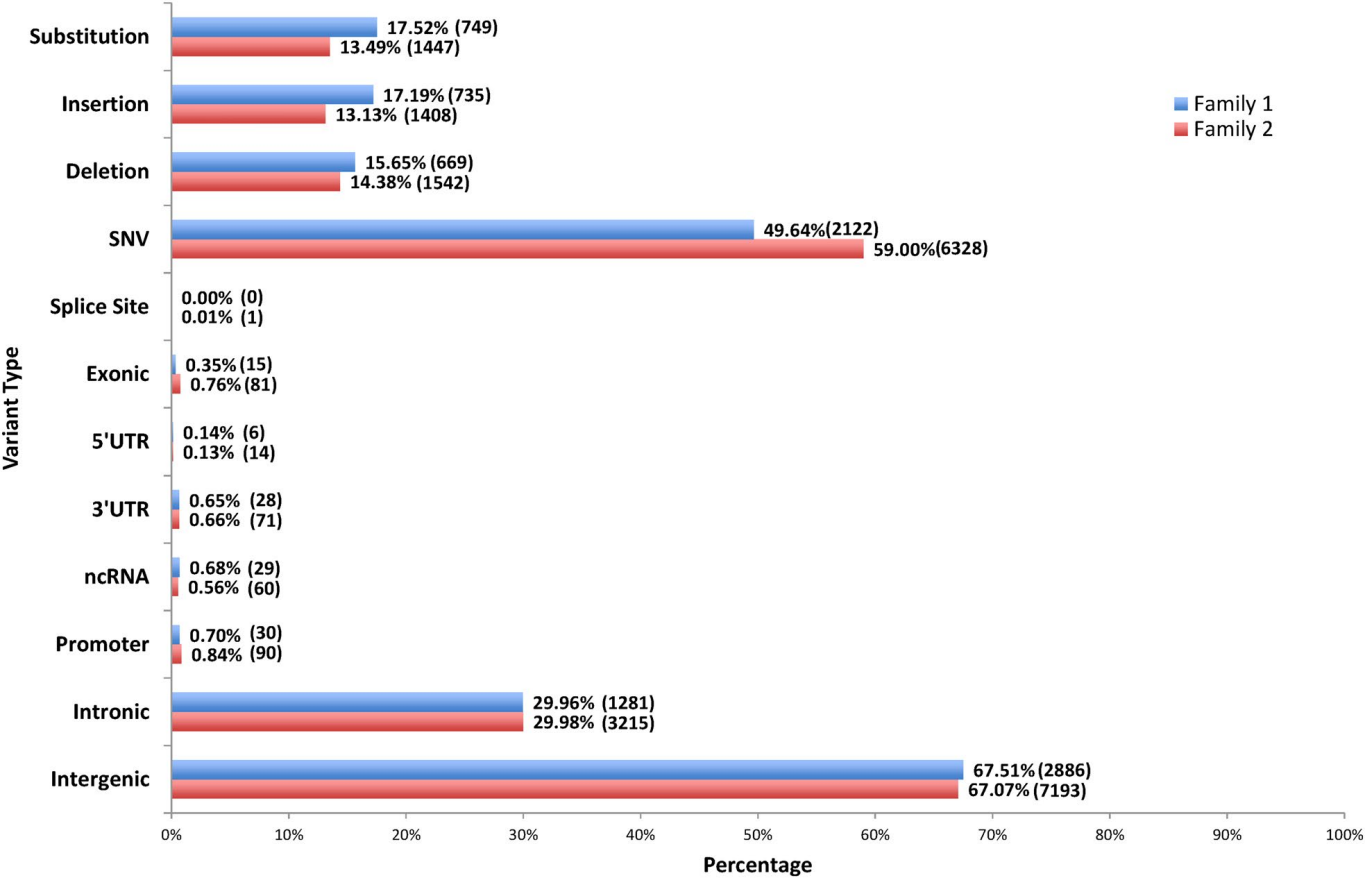
De novo (family 1) and presumed de novo (family 2) variants identified in the affected twin of family 1 (I-2-1) and the affected twin of family 2 (II-2-1)

We classified SNVs, small indels and block substitutions as small sequence changes (SSCs). The identity of de novo (family 1) and *provisional de novo* (family 2) high confidence variations in affected twins shows that majority of variants are inter-genic SNVs and that each individual harbors approximately equal number of block substitutions, insertions and deletions (Figs. 3 and 4). As expected, the exonic variants are relatively rare. The exonic de novo variations identified were annotated with gene information for the two patients (Tables 3 and 4). Of the exonic variants identified, 13 exonic variants in family 1 and 22 exonic variants in family 2 were related to brain function or previously implicated in schizophrenia and related disorders. The patient in family 1 carries four exonic de novo variants in *ZNF595*, a zinc finger protein, two of which are predicted to have a functional impact: one frameshift and one missense mutation. Further, two missense mutations in *MUC3A* (that encode for a transmembrane mucin) and a missense in *OR4C45* (that encode for olfactory receptors) may also contribute to pathophysiology. Interestingly, these variations have not been reported in 1000 genomes as well as CG public genomes and may not be polymorphic. In comparison, the patient from family 2 appears to carry a much larger number of variants. However, in the lack of parental sequences this only represents the differences between the discordant twins. Most (51/82) of these variants in this patient have been reported in 1000 genomes and/or CG public genomes and are considered polymorphic. It leaves 31 variants that may be viewed as *provisional somatic de novo*. The genes affected by missense changes include some that are compatible with the pathophysiology of schizophrenia. These include *NOTCH2, OR2T2, ZNF717, MUC4, MUC6, OR4C3, UBTFL1, TAS2R46* and *KRT6B* (Table 4). However, we note that although these results are appealing, additional validation will be needed to establish a precise role for these genes in the etiology of schizophrenia.

#### De novo mutational features in twins affected with schizophrenia: CNVs

The genome sequences of the six individuals were used to identify individual specific copy number variations as compared to NCBI Build 37. Table 5 shows the distribution of observed CNVs in MZ twins representing the two families. This data allowed classification of CNVs representing inherited (present in at least one parent) and de novo (not present in either parent and also not present in co-twin) for family 1 and unshared between twins in family 2. Of the rare CNVs that were not shared between twins, some represented inherited and others non-inherited and somatic de novo events that must have occurred during independent mitosis following their separation from each other (Tables 6 and 7). In family 1, five of the seven unique CNVs were also not found in either parent. Discordant CNVs between twin pairs were annotated and are presented in Tables 6 and 7, for the affected twin from family 1 and 2, respectively. Three genes overlapped unique CNVs in family 1. The *PDSS1*, and *LOC642131* genes represented somatic de novo events, as they were not present in either parent while *CES1* that was present in one parent was viewed as inherited. Although the affected twin of family 2 also carried five CNVs that were not present in her unaffected twin, these regions did not directly overlap any genes.

#### De novo mutational features in twins affected with schizophrenia: SVs

The complete genome sequences of the six individuals were also used to identify individual specific deletions, tandem duplications, distal duplications, inter-chromosomal variations and inversions in comparison to NCBI Build 37. They were grouped as structural variants (SVs). The SVs ranged from 50 bp to 218 Mb in length. Most of the SVs in family 1 (total 138) and 2 (total 141) represented deletions (78 and 86%), followed by tandem duplications (6 and 10%) and distal duplications (6.5 and 3.5%). Also, we found one inter-chromosomal move involving a translocation of *CDC27* from chromosome 2 to 17 in the patient in family 1 (Additional file 1: Figure S1).

Further, we characterized the genomic details of individual SVs that were unique to the affected twins of the two families (Additional file 1: Table S1 and S2). For family 1 where parental sequences are available we were able to eliminate familial events and concentrate on somatic de novo events. We note that these changes have affected two genes (*ANKS18* and *CLCN5*) via deletion, four genes (*LOC285768, NTM, SNORD115*-*29* and *GZMM*) due to tandem repeats and a 5 Mb tandem duplication on chromosome 1 (825765) affecting 101 genes. Also, it was possible to identify SV differences between twins in family 2, but in the absence of parental sequences they could not be characterized as familial or somatic de novo as in family 1. Consequently, the SV differences between twins in family 2 are viewed as *provisional somatic* de novo. As in family 1 they affected 47 genes by deletion, seven genes at seven sites by tandem duplication along with a 3 Mb tandem duplication on X (52886720) overlapping 39 genes and 2 genes by distal duplication. Also, the genomes carried rare (two or three) inversions, both twin pairs shared them with their co-twin and all inversions were inherited in family 1. Finally, the sharing of CNVs and SVs between any two individuals is directly correlated with their genetic relatedness; high but not 100% between monozygotic twins, low across unrelated individuals and intermediate between a parent and an offspring, as expected.

#### Independent confirmation of NGS results

**Fig. 5:**
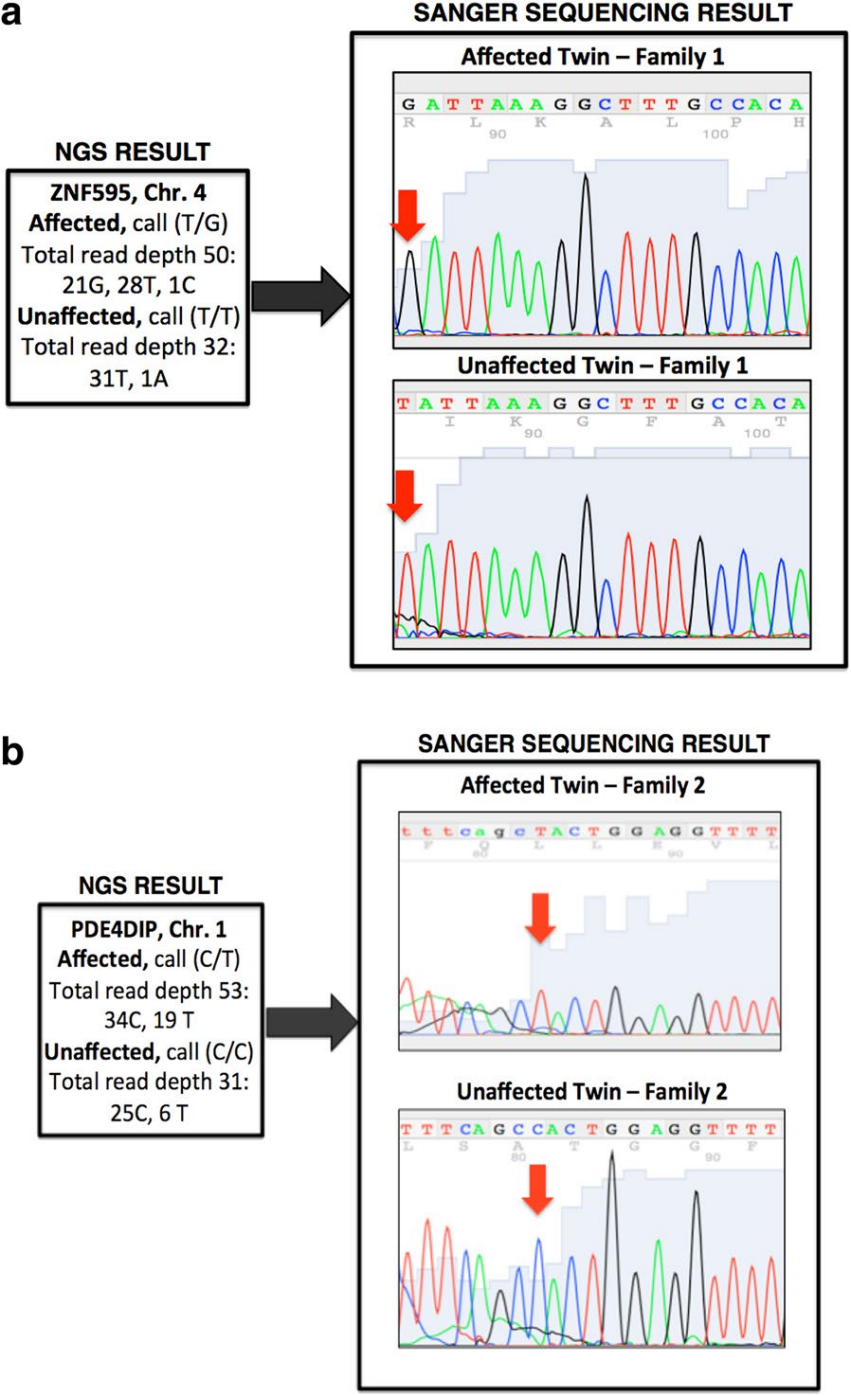
**a** Confirmed variant between monozygotic twins in family 1 in the 4th exon of the ZNF595 gene. This variant changes isoleucine to serine. **b** Confirmed variant between monozygotic twins in family 2 in the 13th exon of the PDE4DIP gene. This variant changes Tryptophan to a premature stop codon

We sought to confirm a random sample of CNVs, SVs and SSCs by independent experiments involving Real Time PCR for confirmation of CNVs and SVs (Table 9) and sanger sequencing for confirmation of SSCs (Fig. 5a, b). They represented 10 randomly chosen structural rearrangements and 30 randomly chosen SSCs, specifically 4 CNVs, 6 SVs and 30 SSCs (two of which failed sanger sequencing). Table 9 outlines the CNVs and SVs that were assessed by Real Time PCR and includes the predicted identity of the variants from sequencing. Each Real Time PCR assay identified the number of copies of each segment of DNA interrogated, which are reported in Table 9. Further, the Real Time PCR for CNVs and SVs using blood DNA confirmed 5/10 variants, three of which were also confirmed on the DNA from buccal swabs from the same individuals. Many of the SSC findings represented shared peaks that may have resulted from expected mosaicism resulting from somatic de novo events during different stages of ontogeny. Given the early differentiation of the germ layers during development, we would expect tissue specific somatic variation to exist, which is compatible with only some of the variation between twins being identified in both blood and buccal tissues in the same individual.

**Table 9:** Results of real time quantitative PCR analysis on 10 CNV/SV regions predicted by NGS to be unshared between twins

Gene/region	Chr	Start	End	Size (bp)	Family	Type	NGS prediction	Real time result in affected twin	Difference between twins?	Buccal DNA
GABRD	1	825765	5726936	4,901,171	1	SV	Increase	2.62	✔	Yes (2.65)
CLSTN2	3	139670597	139674454	3857	2	SV	Increase	1.16	✔	No (1.64)
OPRM1	6	154446930	154447253	323	2	SV	Decrease	2.15	✗	N/A
STX1A	7	66193913	73382120	7,188,207	1	SV	Decrease	2.75	✔	No (1.98)
12q24.21	12	114521470	114529470	8000	2	CNV	Increase	1.33	✔	Yes (0.82)
ANKS1B	12	99793948	99802771	8823	1	SV	Decrease	1.9	✗	N/A
15q11.2	15	22422114	22492114	70,000	1	CNV	Increase	2.69	✔	Yes (2.58)
CES1	16	55841801	55855801	14,000	1	CNV	Increase	1.56	✗	N/A
16p11.2	16	34467150	34515150	48,000	1	CNV	Increase	1.8	✗	N/A
PLTP	20	44535505	44535941	436	1	SV	Decrease	2.1	✗	N/A

### Genetic differences between MZ twins and their discordance for schizophrenia

The results also allow us to assess any involvement of genetic differences between MZ twins in the development of schizophrenia using a threshold model [18]. It assumes that both members of the monozygotic discordant MZD twins will carry family specific genetic predisposition for the disease and rare ontogenetic additional changes in the affected twin will add to the predisposition that will be sufficient for the onset of the disease. This model is testable given near complete coverage of genetic changes in the affected as well as unaffected members of the two MZD twin pairs. It begins with development of a list of all genes that are affected by a variety of mutational mechanisms in four members of the two MZD twin pairs. This list is rather comprehensive and includes all variants, some validated and others not validated. We have used this list to assess the effect of these differences using ingenuity pathway analysis (IPA). It moves the analysis from a focus on affected genes to affected pathways.

**Fig. 6:**
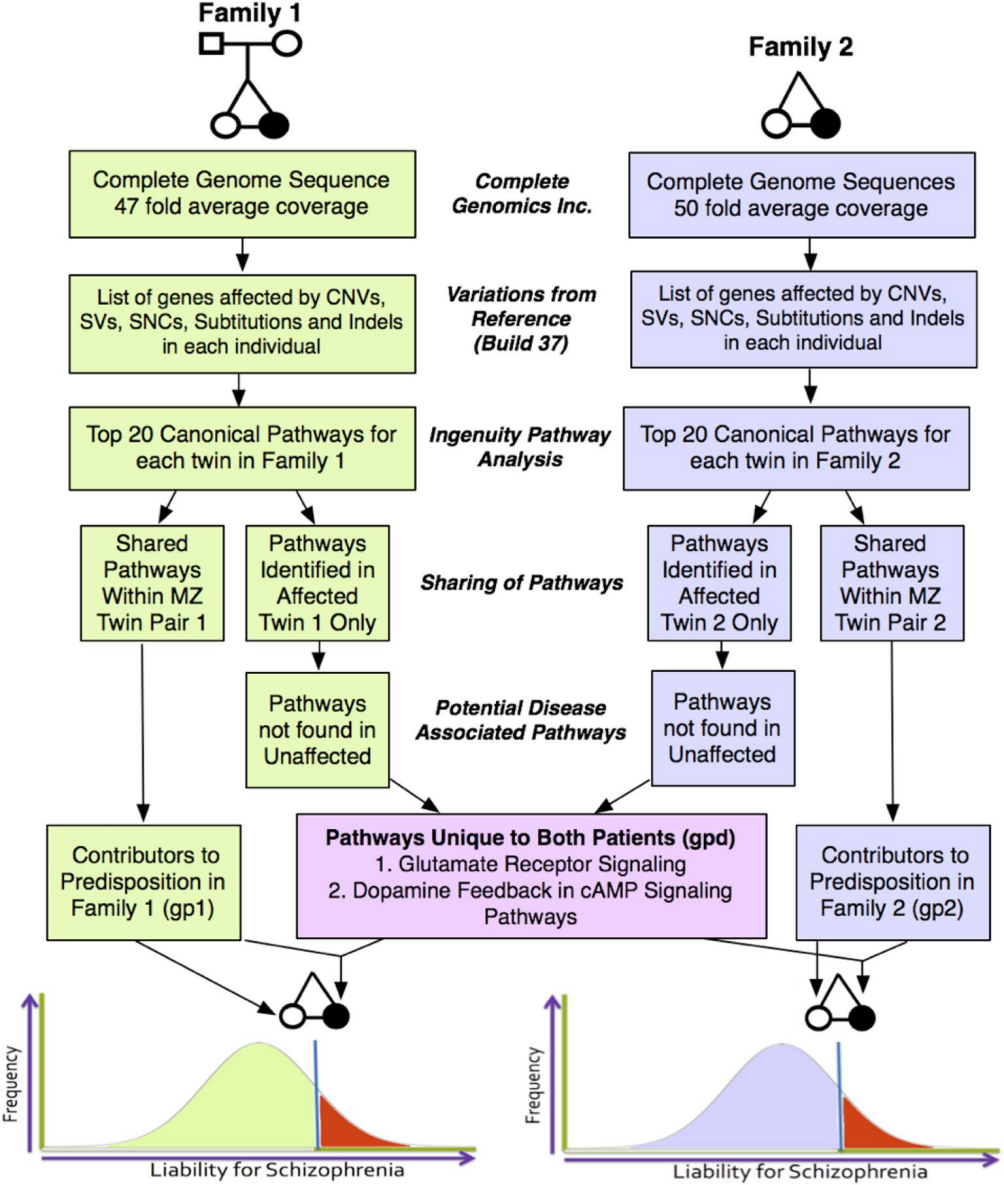
Analysis of ingenuity pathway results

**Fig. 7:**
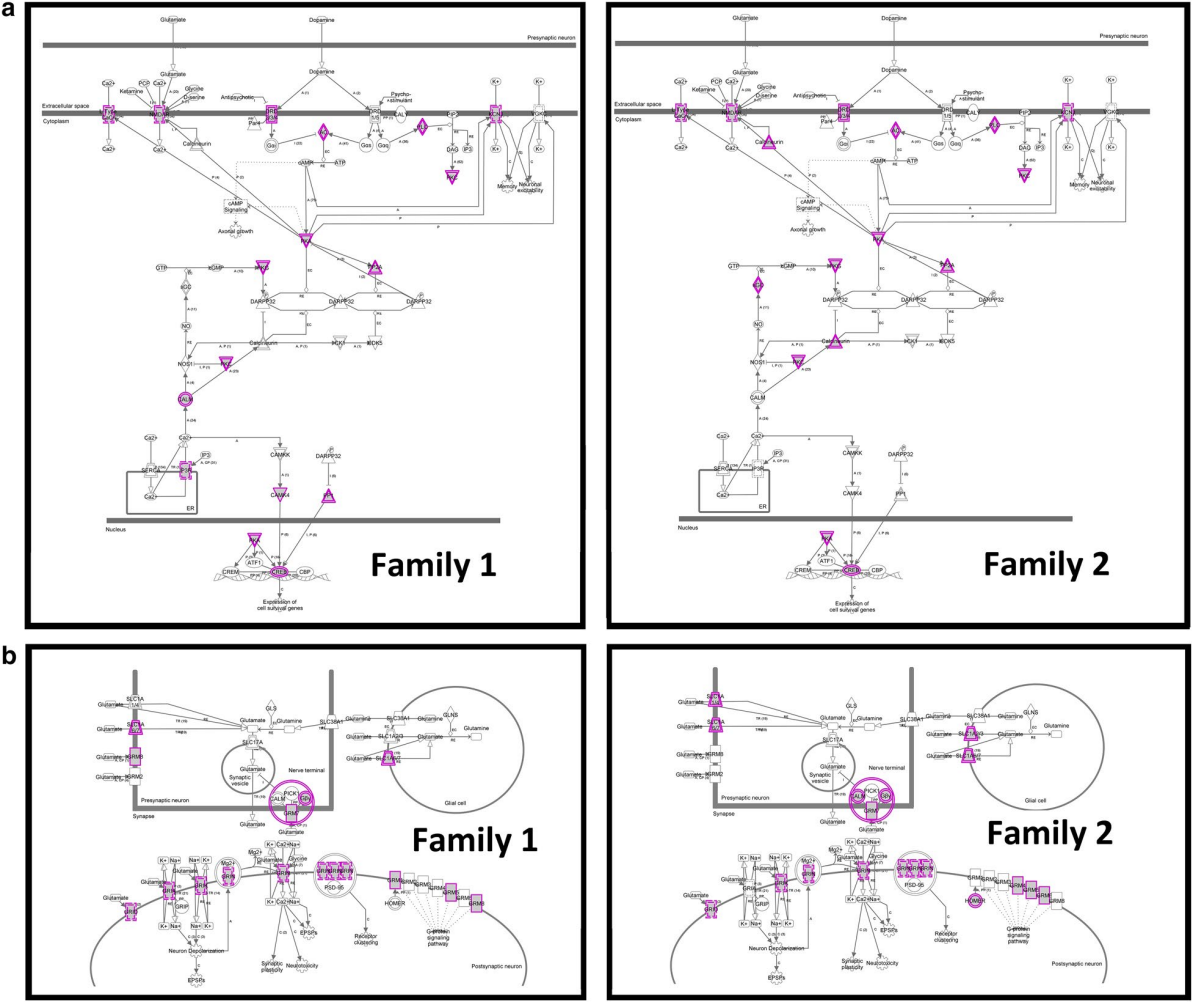
**a** Dopamine feedback in cAMP signaling pathway identified using ingenuity pathway analysis (IPA). Genes that were unique to each affected twin were used to generate canonical pathways of interest. This pathway emerged independently in both affected twins. This pathway was also not found to be enriched in unaffected twins in the study. Purple represents genes harboring a unique high confidence variant in each respective patient (family 1, *p* = 2.28E−04; family 2, *p* = 5.84E−03). **b** Glutamate pathway identified using ingenuity pathway analysis (IPA). Genes that were unique to each affected twin were used to generate canonical pathways of interest. This pathway emerged independently in both affected twins. This pathway was also not found to be enriched in unaffected twins in the study. Purple represents genes harboring a unique high confidence variant in each respective patient (family 1, *p* = 1.46E−03; family 2, *p* = 3.88E−05)

Table 10 shows the top 20 individual specific canonical pathways in two affected individuals belonging to the two families. Interestingly, these pathways are not random. Figure 6 incorporates our strategy in assessing the nature of the threshold model in the two patients [36]. Of the top 20 pathways in patient 1 and patient 2, 10 are shared between them (Additional file 1: Figure S4). Also, 8 of the 10 pathways shared by the two patients are present in either of the two unaffected members of the two twin pairs. It leaves two pathways that may be viewed as highly specific to the two patients (Fig. 6). They represent dopamine-DARPP32 feedback in cAMP signaling (Fig. 7a, family 1, *p* = 2.28E−04; family 2, *p* = 5.84E−03) and glutamate receptor signaling (Fig. 7b, family 1, *p* = 1.46E−03; family 2, *p* = 3.88E−05). We note that 13 and 29 genes of the two pathways respectively are affected in the patient of family 1. The corresponding numbers for the patient in family 2 are 15 and 23 genes for the two pathways, respectively (Table 11). Although few of the mutated genes are patient specific, the two patients share a large number of affected molecules in glutamate receptor signaling as well as dopamine-DARPP32 feedback in cAMP signaling pathways. Further, we note (Table 10) that four of the top five pathways in patient of family 1 and three of the top five canonical pathways in patient of family 2 are relevant to neural functions and have the potential to contribute to the disease in a threshold model discussed in the next section.

**Table 10:** Top 20 canonical pathways identified by ingenuity pathway analysis (IPA) in the affected twin of each family

Canonical pathways—family 1 affected (1A)	Shared with	*p* value
1. CREB signaling in neurons	2A, 2U	0.0000041687
2. Neuropathic pain signaling in dorsal horn neurons	2A, 1U, 2U	0.0000083176
3. Axonal guidance signaling	2A, 1U	0.0000630957
4. Cellular effects of sildenafil (Viagra)	2A, 1U	0.0001479108
5. Role of NFAT in cardiac hypertrophy	–	0.0001513561
6. Dopamine-DARPP32 feedback in cAMP signaling	2A	0.0002290868
7. Synaptic long term depression	2A, 1U, 2U	0.0002398833
8. Wnt/Ca+ pathway	–	0.0003630781
9. Synaptic long term potentiation	2A, 2U	0.0008511380
10. PPARÎ±/RXRÎ± activation	2U	0.0011748976
11. Gap junction signaling	–	0.0013803843
12. Glutamate receptor signaling	2A	0.0014791084
13. 14-3-3-mediated signaling	–	0.0020417379
14. Netrin signaling	2A, 1U, 2U	0.0022908677
15. Leptin signaling in obesity	–	0.0024547089
16. Nitric oxide signaling in the cardiovascular system	2A, 1U	0.0024547089
17. Hepatic cholestasis	2U	0.0026915348
18. Uracil degradation II (reductive)	2U	0.0029512092
19. Thymine degradation	2U	0.0029512092
20. Melatonin signaling	1U	0.0033113112

Canonical pathways—family 2 affected (2A)	Shared with	*p* value
1. Sperm motility	1U	0.0000053703
2. Glutamate receptor signaling	1A	0.0000389045
3. Neuropathic pain signaling in dorsal horn neurons	1A, 2U, 1U	0.0000575440
4. Cellular effects of sildenafil (Viagra)	1A, 1U	0.0002238721
5. Nitric oxide signaling in the cardiovascular system	1A, 1U	0.0002884032
6. Synaptic long term depression	1A, 2U, 1U	0.0003235937
7. CREB signaling in neurons	1A, 2U	0.0003630781
8. Synaptic long term potentiation	1A, 2U	0.0005888437
9. Phospholipase C signaling	–	0.0008709636
10. Netrin signaling	1A, 2U, 1U	0.0010964782
11. G-Protein coupled receptor signaling	2U	0.0012589254
12. Î±-adrenergic signaling	–	0.0016595869
13. Protein kinase A signaling	2U, 1U	0.0023442288
14. nNOS signaling in neurons	–	0.0040738028
15. Huntington’s disease signaling	–	0.0042657952
16. Dopamine-DARPP32 feedback in cAMP signaling	1A	0.0058884366
17. Cardiac Î^2^-adrenergic signaling	2U	0.0067608298
18. Calcium signaling	2U	0.0077624712
19. Breast cancer regulation by Stathmin 1	–	0.0087096359
20. Axonal guidance signaling	1A, 1U	0.0089125094

*1A* Family 1—affected twin, *1U* Family 1—unaffected twin, *2A* Family 2—affected twin, *2U* Family 2—unaffected twin

**Table 11:** Genes affected in two pathways (glutamate receptor signaling and dopamine feedback in cAMP signaling) unique to the two unrelated schizophrenia patients representing family 1 and family 2

Pathway	Family 1: genes in pathway		Family 2: genes in pathway		Common to both affected twins
	Number of genes affected	Identity	Number of genes affected	Identity	
Dopamine feedback in cAMP signaling	29	PRKG2, PPP2R1U, GUCY1A3, PRKAG2, ADCY3, PRKCB, PRKAR1B, KCNJ12, ADCY2, PRKCZ, ADCY8, PLCL1, CACNA1D, DRD3, PLCB1, GUCY1A2, EP300, PRKG1, PPP3CA, CACNA1A, GRIN2U, PLCG2, KCNJ3, PRKCA, PLCH2, CACNA1C, PLCL2, PLCZ1, KCNJ10	23	PLCB1, PRKG2, PRKG1, PPM1L, CAMK4, PLCE1, PRKAG2, PPP2R2C, ADCY10, PPP1R14C, GRIN2A, CREB5, PRKCA, CACNA1C, PRKCB, KCNJ12, ITPR1, PRKAR1B, PRKCI, PRKCZ, ADCY8, PRKCG, DRD3	12 (PRKG2, PRKAG2, PRKCB, PRKAR1B, KCNJ12, PRKCZ, ADCY8, DRD3, PLCB1, PRKG1, PRKCA, CACNA1C)
Glutamate receptor signaling	13	GRIA1, GRIK2, GRID1, GNB1, GRIN2U, SLC1A7, GRIK1, GRIK4, GRM7, GRM1, GRM5, GRM8, GRIK3	15	GRID1, GNG7, CAMK4, SLC1A2, HOMER1, SLC1A7, GRIK1, GRIN2A, GRM4, GRIK4, SLC1A1, GRM7, GNG2, GRM5, GRM6	6 (GRID1, SLC1A7, GRIK1, GRIK4, GRM7, GRM5)

## Discussion

### Individual human genomes contain extensive variability

In general, the nature and distribution of variations observed in this study follow the results on 1000 genomes data and expected distributions based on chromosome size (Figs. 3 and 4). It follows that each individual harbors hundreds of *rare* variants [37]. The individual genomes have on average, 3.6 million SNVs, 344 thousand indels and 717 large deletions [37] that includes somatic as well as germline de novo variations. However, the rate of somatic de novo mutation appears to vary and may be family specific [38]. Unfortunately, this variability particularly caused by somatic mutations is not always easy to discern. Not surprisingly, almost all de novo mutations reported in the literature are based on germline events. These estimates are much lower than our results that focus on somatic de novo events representing individuals of different ages. Interestingly, our results follow a recent report that found that the rate of de novo somatic mutations in humans and mice is almost two orders of magnitude higher than the germline mutation rate and that both mutation rates are significantly higher in mice than humans [39]. It also adds that somatic de novo mutations are highly variable, may depend on tissue and age of the individual, and could add to predisposition for disease.

Studies of twin sequencing differences in the literature have noted validation rates of variants as low as 1/15 [40]. Often it is attributed to mosaicism resulting from de novo somatic events. In addition, validation rates for data from the 1000 genomes project averaged a validation rate of 1.8%, and similar to our study, large SVs were easier to validate than other types of variation. Our analysis also focused on the use of higher quality variant calls as was done in the 1000 genomes pilot study to evaluate the likelihood of a candidate variant call being a real event [37]. The results show that individual genomes harbor extensive variability and that this variability can be measured both within and between generations.

### MZ twins are genomically different

Results included in this report show that the genome sequences of pairs of MZ twins although very similar, are not identical. It follows a number of recent reports on identical twins [41–45]. Although likely to be a conservative estimate, Krawczak et al. has predicted that there exists at minimum an average of > 1.3 SNVs discriminating MZ twins in each tissue type [46]. The results of one forensic study found DNA sequence differences between identical twins that appear to reflect mosaicism in that the newly arisen allele was generally only found in a small fraction (approximately 20% of the cells assayed) as estimated by sanger sequencing results [41]. Many reports have now identified genetic and epigenetic differences between identical twins [6–8]. For example, Bruder et al. [4] reported that all of 19 MZ twins studied differed in CNVs [47] and Forsberg et al. confirmed 10 post-twinning CNV mutations in 159 MZ twin pairs [48]. In addition, many post-twinning single nucleotide mutations have also been reported [42–45]. It is likely that older twin pairs will have accumulated more somatic de novo mutations over time [45] and this will be expected to affect the degree of somatic mosaicism across tissues [49]. A recent report that identified somatic mutations at the base-pair level in monozygotic twins found two de novo somatic mutations that appear to have occurred early in embryonic development, suggesting that early development may be enriched for de novo change [12].

### De novo changes may add to genetic liability in schizophrenia

Schizophrenia is a multifactorial and highly heterogeneous disorder and may involve both genomic and environmental contributions [50]. Population and family studies have implicated a number of mutational mechanisms (SSCs, CNVs and SVs) and many genes of small effect in the causation of this disease [51–53] with no single gene affected in all cases. Conceptually, an accumulation of mutations affecting a number of genes affecting disease specific pathways may establish a genetic liability gradient for schizophrenia where a critical threshold is required for the manifestation of the disease state [54]. Further, the underlying genetic liability is expected to be higher in families with schizophrenia patients. At the individual level, this liability will be much higher in the related family members. In the case of MZ twins where one is affected with the disease, the affected member is expected to have acquired additions to the liability scale via gene mutations and/or environmental factors that contribute to threshold crossing and thus development of disease. It remains an attractive hypothesis but has remained untested, primarily because of the lack of appropriate data. Also, threshold models are often assessed at the level of the population. This study is unique in that it applies this model to cases representing individual patients representing MZD twins along with a genetically matched control.

The current study, with complete genome sequences of pairs of MZD twins is novel and offers a number of advantages. First, it focuses on individual patients and matches the patient with her unaffected monozygotic twin. The consequence is that it drastically reduces genetic heterogeneity between a case and a control. Second, it focuses on the cause of the disease in highly selected and clinically defined individual patients rather than grouping together heterogeneous cases of schizophrenia. Third, the availability of complete genome sequences allows for identification of almost all forms of genetic mutations per individual. Fourth, the identification of all affected genes in a patient allows for further identification of canonical pathways that may be affected by individual specific gene mutations. The threshold model argues that a pathway may be affected by mutations involving different sets of genes in different individuals. More importantly, the two members of a MZD twin pair would share a common genetic liability for the disease with the affected twin acquiring yet additional defects causing it to cross the disease threshold on the liability continuum and develop schizophrenia.

We have assessed the genomic data obtained in our experiments to evaluate the threshold model of schizophrenia (Fig. 6) [36]. This analysis has yielded a set of canonical pathways for four individuals representing two twin pairs. The top 20 pathways identified in four members of the two twin pairs are given in Table 10. We focused on affected pathways that were shared or not shared in the two MZD twins. Within twin pair sharing of pathways was viewed to represent “genetic predisposition” and were considered to not be sufficient to cause the disease; we labeled these predisposing pathways as GP1 in family 1 and GP2 in family 2. Naturally, the composition of GP1 and GP2 (GPN across n pairs of twins) is expected to differ between unrelated pairs. Further, we selected pathways that were unique to the two patients in this study. We argue that these additional defects were needed to cross the threshold and manifest the disease; we labeled these disease-causing pathways GPD. Once again, the composition of GPDs may vary across families and includes all variants, some validated and others not validated. In this analysis, we have identified the same GPDs in both families which involved two pathways; dopamine feedback in cAMP signaling pathway (Fig. 7a) and glutamate receptor signaling (Fig. 7b). Interestingly, a number of pathways identified in the genetic predisposition lists (GP1 and GP2) have been previously implicated in this heterogeneous disease. These include the dopamine feedback in cAMP signaling pathway [55–57], glutamate receptor signaling [58, 59], CREB signalling in neurons [60], axonal guidance signalling, Netrin signaling [61] and nNOS signalling in neurons [62]. Independent identification of known schizophrenia related defects from the affected twin supports the argument that the observed differences between MZD twins are not random sequencing artifacts. More important, they offer a logical explanation for the discordance of the two MZ twins with long term discordance for schizophrenia.

#### Dopamine feedback in cAMP signaling in schizophrenia

The dopamine feedback in cAMP signalling pathway is one of the pathways that has emerged independently in both schizophrenia-affected twins in this study (Fig. 7a). This pathway has been shown to be associated with psychiatric disorders due to the fact that it has critical function in integrating dopaminergic and glutamatergic signalling, and in turn affecting striatal function and plasticity [56, 57]. The leading theory to account for the pathophysiology of schizophrenia involves an excess of the neurotransmitter dopamine, either through excess production or postsynaptic dopamine over-activity possibly mediated by increased receptor density [55]. Recent reports reinforce the importance of dopamine in schizophrenia, including strong associations with *DRD2* (a target of many antipsychotic drugs) [16]. The results support the contention that the defects associated with this pathway have the potential to contribute to schizophrenia in the two patients studied.

#### Glutamate receptor signalling in schizophrenia

A glutamate receptor-signalling pathway is the second pathway that is found in the two patients studied (Fig. 7b). The significance of this pathway in this disease is backed by the underlying neurochemical basis of schizophrenia that includes a hypofunctional glutamate system [63]. Many genes associated with glutamateric neurotransmission have been previously implicated in schizophrenia, including *GRM3, GRIN2A, SRR* and *GRIA1* [16]. Further, morphological alteration of dendrites of glutamatergic neurons in the cerebral cortex of schizophrenia-affected individuals have been reported [64] suggesting a role for glutamate signalling in the etiology of schizophrenia. Interestingly, schizophrenia patients show enriched de novo mutations in genes regulating the postsynaptic density at glutamatergic synapses [58]. Furthermore, small de novo mutations were found to be overrepresented among glutamatergic postsynaptic proteins [59] and genes harbouring detrimental de novo mutations were reported to be enriched in networks affecting protein interactions [65]. In addition, a recent study investigating rare mutations in exonic regions of genes implicated in schizophrenia and autism spectrum disorder revealed that post-synaptic glutamate receptor complexes are key molecular mechanisms associated with schizophrenia and ASD [66]. We propose that the two pathways described above have the potential to at least partially contribute to the disease in these two patients. Also, given that the two pathways were identified from two patients from two unrelated families, they may represent recurring defects in this disease for a subset of patients.

### Disease model: somatic de novo mutations interject in the threshold model of schizophrenia

The results included in this report also show that somatic de novo mutations may play a significant role in the development of schizophrenia. These observations are backed by extensive literature that argue for the existence of somatic mutation in our genomes [12, 41, 67, 68]. In this model, MZD twins with the disease are argued to have acquired additional de novo errors onto an already existing background of genetic predisposition and thus contributing to disease liability. These de novo errors must involve somatic mutations during ontogeny [3, 69]. Rare post-zygotic mutations are likely to have been underestimated in the past. Mutations that occur later in development are likely to be seen in very low frequency and simply look like sequencing errors in any analysis [70]. The high-quality results, publicly accessible for further analysis, support the proposition that de novo alterations may play a role in the manifestation of schizophrenia. The mutations identified are predominantly seen in genes already known to be implicated in schizophrenia and related disorders. They help highlight major processes affected in specific patients. They argue for extensive heterogeneity in disease processes. For the two patients studied here, they identify aberrations in dopamine feedback and glutamate receptor signalling pathways.

It has not escaped our mind that this model will account for a variety of results and observations reported in the extensive literature. For example, it is possible to override the liability scale established by small effects of a number of genes with large effects on the disease phenotype [24, 71]. Some of these may result from de novo mutation in MZD or singleton cases [3, 72, 73]. Also, these changes must be present in the brain. In fact, de novo mutations are more likely to occur in schizophrenia patients than unaffected siblings [74] and damaging de novo mutations in schizophrenia patients were found to disrupt genes regulating neurogenesis in the postmortem human brain [21]. Interestingly, de novo mutations are now known to be rather pervasive in the brain [75, 76]. They may represent a unique source of neuronal diversity and individual differences. We also note that by its very nature, the effect of a de novo mutation may or may not be always apparent depending on the organ system affected and the degree of mosaicism. The impact of such mutations on disease causation is being recognized and deserves intensive investigation [77]. Finally, individual specific exceptional comprehensive genome-wide results included in this report provide a promising approach to the understanding of schizophrenia and may apply to other related disorders.

## Conclusions

The complete genome sequences of monozygotic twins discordant for schizophrenia have allowed for direct identification of patient specific somatic de novo mutations that may augment the threshold of liability and cause this neurodevelopmental disease. The results also identify variants in the unaffected twin as fragment of genetic predisposition that are not sufficient to manifest the disease. Together they support two propositions. First, somatic de novo mutations, that are pervasive in the brain, may play a role in the development of schizophrenia and second, discordance of monozygotic twins for schizophrenia and related neurodevelopmental diseases in some families may be influenced more heavily by genetic, rather than environmental effects.

## Additional file

**Additional file 1.** Additional Figures S1–S4 and Tables S1, S2.

## Abbreviations

CNV: copy number variant
GP: genetic predisposition
GPD: genetic predisposition leading to disease
IPA: ingenuity pathway analysis
MZ: monozygotic
MZD: monozygotic discordant
SNV: single nucleotide variant
SSCs: small sequence changes (SNVs, indels, block substitutions)
SV: structural variant
