# Supplementary material for: Post-zygotic genomic changes in glutamate and dopamine pathway genes may explain discordance of monozygotic twins for schizophrenia
Source: Clin Transl Med. 2017 Nov 28;6:43. doi: 10.1186/s40169-017-0174-1 (PMC5704032; doi:10.1186/s40169-017-0174-1)
Supplement: Supplementary file 1 — Additional file 1. Additional Figures S1–S4 and Tables S1, S2. [file 40169_2017_174_MOESM1_ESM.pdf]

Supplemental Tables and Figures

Post-zygotic de novo changes in glutamate and dopamine pathways may explain discordance of monozygotic twins for schizophrenia

Castellani, CA., Melka, MG., Gui, JL., Gallo, AJ. , O'Reilly, RL., Singh, SM

The University of Western Ontario

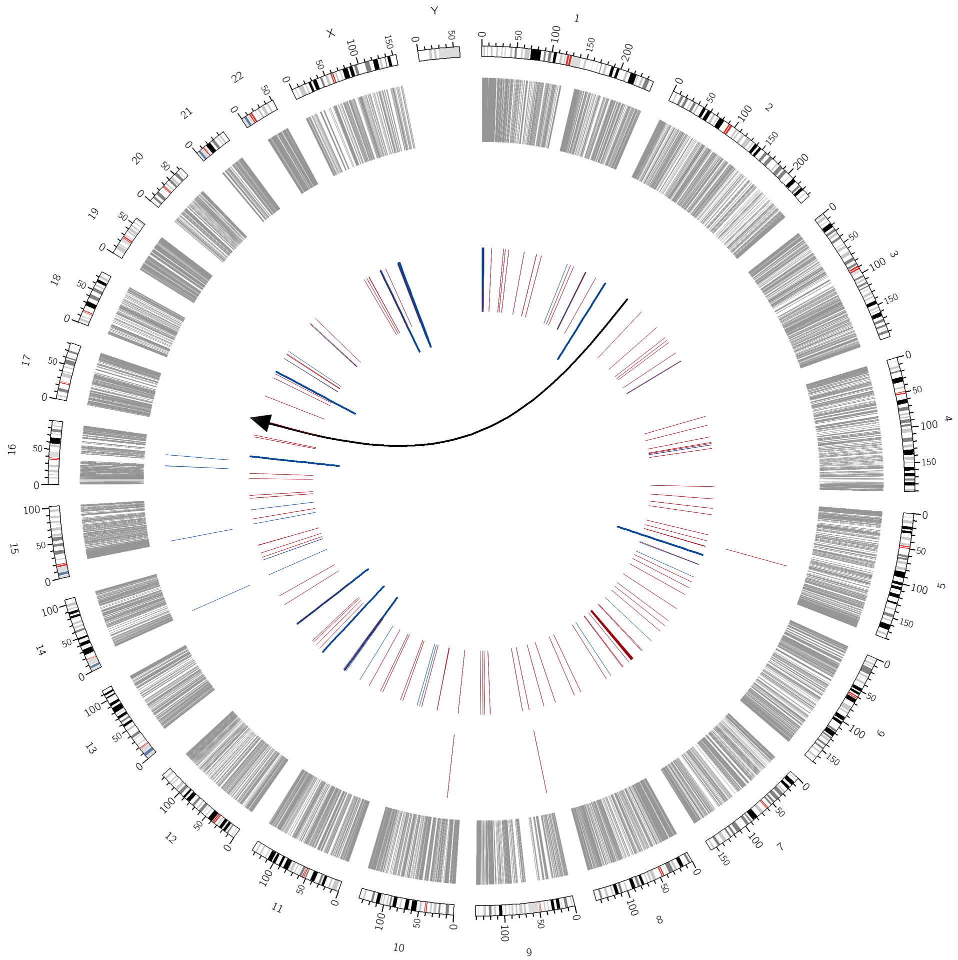

Figure S1-A. Circos plot of high confidence *unique* mutations in the affected member of Family 1. These variations were unshared between twins and not found in either parent. Blue represents a gain and red represents a loss. Outside track=SNVs, Indels, Subs, Middle track=CNVs and Inside track= SVs. The arrow represents an inter chromosomal event.

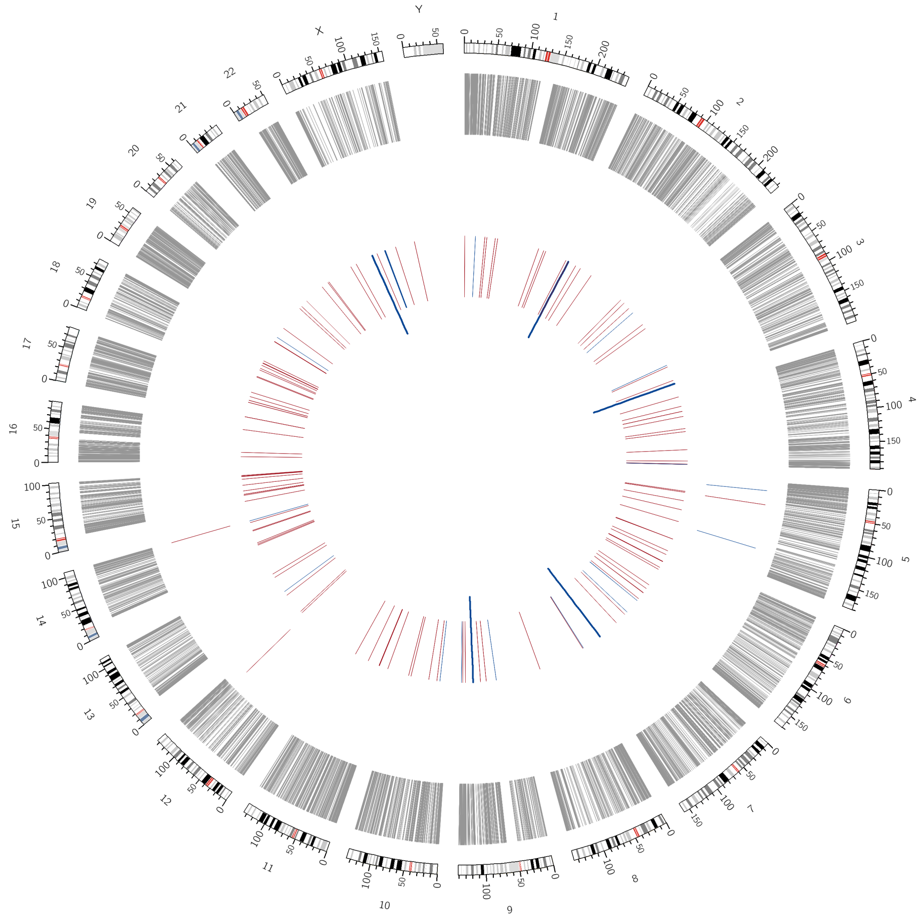

Figure S1-B. Circos plot of high confidence *unique* mutations in the affected member of Family 2. These variations were unshared between twins. Blue represents a gain and red represents a loss. Outside track=SNVs, Indels, Subs, Middle track=CNVs and Inside track= SVs.

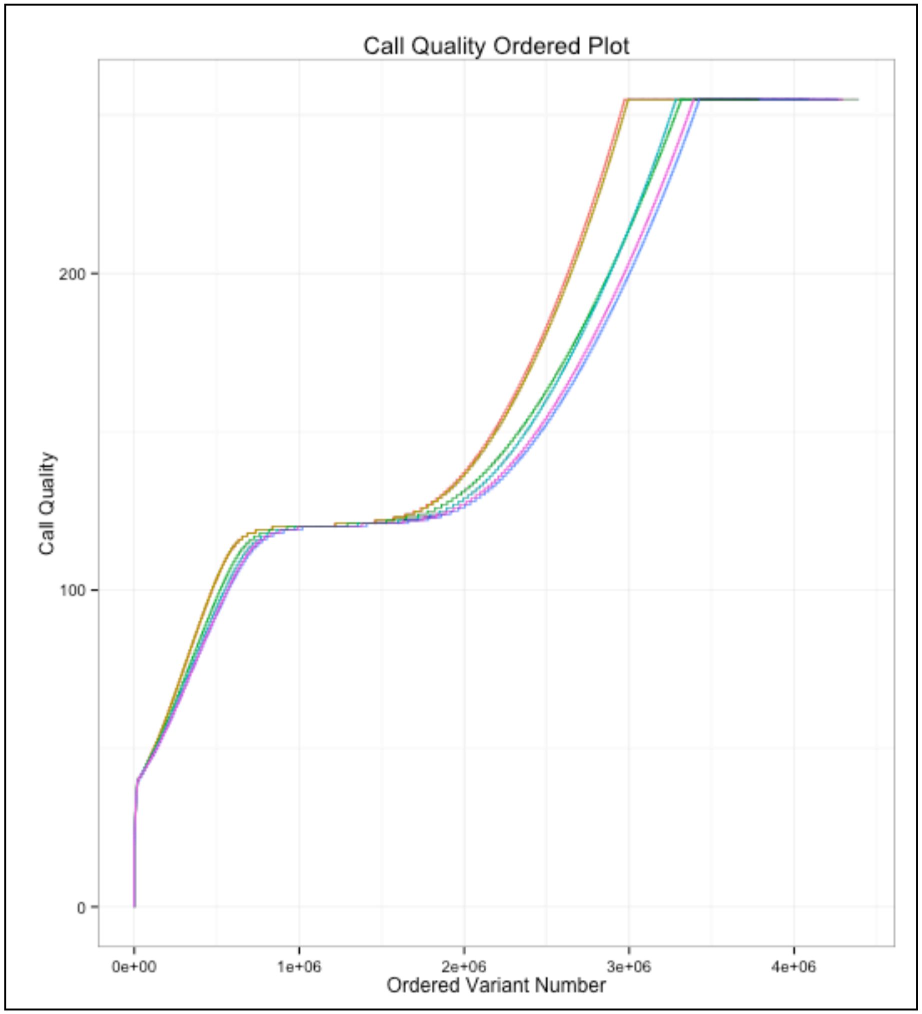

Figure S2. Call Quality of raw variant calls.

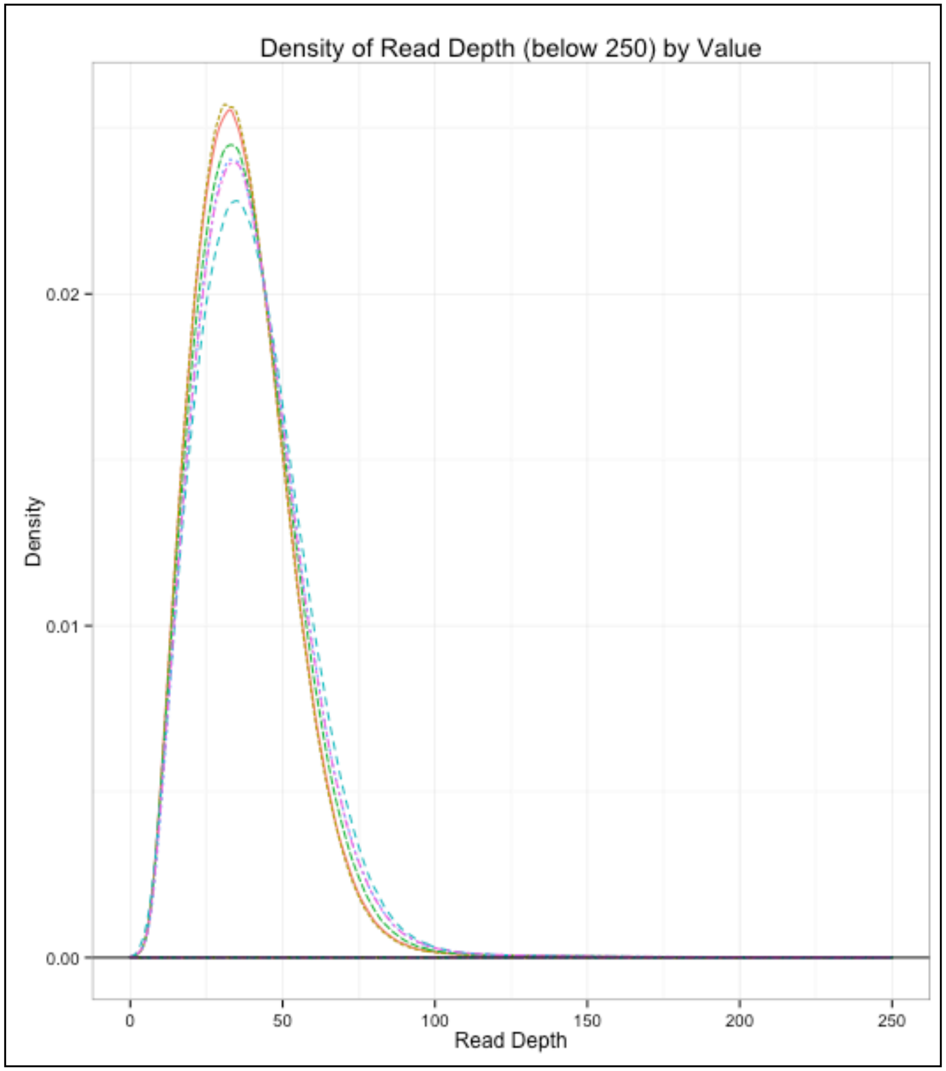

Figure S3. Read Depth Density of raw variant calls. Outliers (above 250) were not included

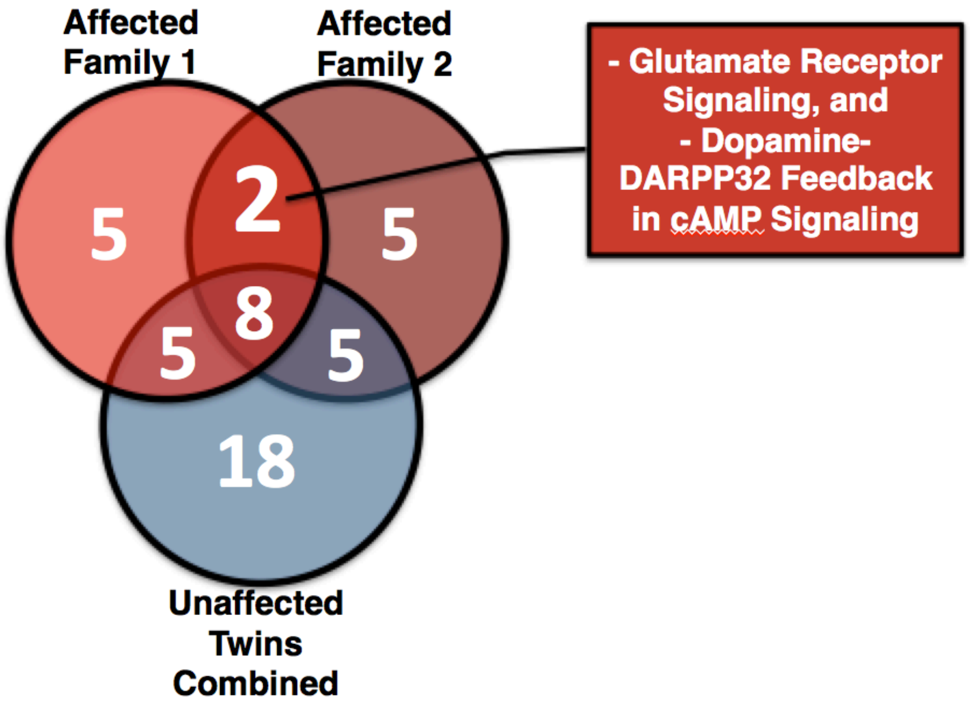

Figure S4. Venn diagram of shared top 20 canonical pathways found to be overrepresented by variants in the sequencing dataset of affected and unaffected twins. The pathways from the unaffected twin in Family 1 and Family 2 have been merged to represent 36 unique pathways (4 shared). The 18 pathways unique to unaffected twins are broken down into 11 from Family 1 and 7 in Family 2.

Table S1. Structural Variants unique to the affected member of Family 1 (I-2-1). Shared identity is based on a 50% reciprocal overlap rule.

Chr	Start	End	Size (bp)	SV Type	Gene(s)	Father	Mother	Inheritance
1	5717300	5725895	8595	Deletion	0	Present	Absent	Inherited
1	5727877	5728315	438	Deletion	0	Present	Present	Inherited
1	26460142	26464816	4674	Deletion	0	Present	Absent	Inherited
1	57242625	57242964	339	Deletion	C1orf168	Present	Present	Inherited
1	62654769	62657187	2418	Deletion	0	Present	Absent	Inherited
1	71237363	71240327	2964	Deletion	0	Present	Absent	Inherited
1	112691792	112704705	12913	Deletion	0	Present	Present	Inherited
1	144894289	144895285	996	Deletion	PDE4DIP	Present	Present	Inherited
1	145026746	145027069	323	Deletion	PDE4DIP	Present	Present	Inherited
1	158867539	158869984	2445	Deletion	0	Present	Present	Inherited
1	238852322	238853265	943	Deletion	0	Present	Absent	Inherited
1	248051492	248057652	6160	Deletion	0	Present	Present	Inherited
2	1801743	1802067	324	Deletion	MYT1L	Present	Absent	Inherited
2	3822800	3823598	798	Deletion	0	Present	Present	Inherited
2	33091738	33095634	3896	Deletion	LOC285045	Present	Present	Inherited
2	182565264	182565592	328	Deletion	0	Present	Absent	Inherited
2	239626179	239627520	1341	Deletion	0	Present	Present	Inherited
3	228847	234481	5634	Deletion	0	Present	Present	Inherited
3	8718847	8722982	4135	Deletion	0	Present	Absent	Inherited
3	16239409	16241210	1801	Deletion	GALNTL2	Present	Absent	Inherited
3	49494287	49494616	329	Deletion	0	Present	Present	Inherited
3	73939035	73939381	346	Deletion	0	Present	Absent	Inherited
4	4568030	4568628	598	Deletion	0	Present	Absent	Inherited
4	28511981	28513202	1221	Deletion	0	Present	Absent	Inherited

4	59428999	59435542	6543	Deletion	0	Present	Absent	Inherited
4	73831249	73831821	572	Deletion	0	Present	Absent	Inherited
4	76743108	76744645	1537	Deletion	0	Present	Present	Inherited
4	80888062	80894107	6045	Deletion	ANTXR2	Present	Present	Inherited
4	90427886	90428672	786	Deletion	0	Present	Absent	Inherited
4	190535505	190538363	2858	Deletion	0	Present	Absent	Inherited
5	668063	668593	530	Deletion	LOC100132605;TPPP	Present	Present	Inherited
5	25959078	25959871	793	Deletion	0	Present	Absent	Inherited
5	42628539	42631218	2679	Deletion	GHR	Present	Absent	Inherited
5	95343326	95343680	354	Deletion	0	Present	Present	Inherited
5	112224253	112226906	2653	Deletion	REEP5	Present	Absent	Inherited
5	126126039	126127716	1677	Deletion	LMNB1	Present	Absent	Inherited
5	127172332	127173282	950	Deletion	LOC728586	Present	Present	Inherited
5	176387587	176390188	2601	Deletion	UIMC1	Present	Absent	Inherited
6	14745281	14745697	416	Deletion	0	Present	Present	Inherited
6	31193512	31194660	1148	Deletion	0	Present	Absent	Inherited
6	51739577	51745615	6038	Deletion	PKHD1	Present	Absent	Inherited
6	95193324	95194338	1014	Deletion	0	Present	Present	Inherited
6	133371501	133371845	344	Deletion	0	Present	Absent	Inherited
6	149409430	149410432	1002	Deletion	0	Present	Absent	Inherited
6	168059226	168059550	324	Deletion	0	Present	Absent	Inherited
7	33898856	33899187	331	Deletion	0	Present	Present	Inherited
7	36441222	36441996	774	Deletion	ANLN	Present	Absent	Inherited

7	66193913	73382120	7188207	Deletion	ABHD11;AUTS2;BAZ1B;BCL7B;C7orf42;CALN1;CLDN3;CLDN4;DNAJC30;FKBP6;FZD9;GTF2IRD2P;LOC100093631;LOC100128031;LOC100131972;LOC100288540;LOC100289307;LOC100289339;LOC442572;LOC644794;LOC729156;MLXIPL;NCF1B;NSUN5;NSUN5C;PMS2L4;POM121;RABGEF1;SBDS;SBDSP;SPDYE7P;SPDYE8P;STAG3L3;STAG3L4;STX1A;TBL2;TRIM50;TRIM74;TYW1;TYW1B;VPS37D;WBSCR17;WBSCR22;WBSCR26;WBSCR27;WBSCR28	Present	Present	Inherited
7	81441673	81442580	907	Deletion	0	Present	Present	Inherited
7	100613739	100614479	740	Deletion	MUC12	Present	Absent	Inherited
7	102800742	102801402	660	Deletion	0	Present	Absent	Inherited
7	155199777	155201525	1748	Deletion	0	Present	Absent	Inherited
8	40774677	40779833	5156	Deletion	0	Present	Absent	Inherited
8	59078464	59078800	336	Deletion	0	Present	Absent	Inherited
8	113085436	113085766	330	Deletion	0	Present	Present	Inherited
8	144056582	144057161	579	Deletion	0	Present	Absent	Inherited
9	6700565	6710685	10120	Deletion	0	Present	Present	Inherited
9	24502071	24519103	17032	Deletion	0	Present	Present	Inherited
9	110018286	110020857	2571	Deletion	0	Present	Absent	Inherited
9	124411502	124411804	302	Deletion	DAB2IP	Present	Absent	Inherited
9	129492472	129492919	447	Deletion	0	Present	Absent	Inherited
9	135622738	135627436	4698	Deletion	C9orf98	Present	Absent	Inherited
10	22081665	22082212	547	Deletion	DNAJC1	Present	Absent	Inherited
10	78346590	78351578	4988	Deletion	0	Present	Absent	Inherited
10	78800911	78801230	319	Deletion	KCNMA1	Present	Absent	Inherited
10	119634283	119634614	331	Deletion	0	Present	Absent	Inherited
11	5784571	5809284	24713	Deletion	OR52N1;OR52N5	Present	Absent	Inherited
11	12152558	12152882	324	Deletion	MICAL2	Present	Present	Inherited
11	63698909	63701654	2745	Deletion	0	Present	Present	Inherited

11	69400843	69401405	562	Deletion	0	Present	Present	Inherited
11	102751130	102752828	1698	Deletion	LOC100288111	Present	Absent	Inherited
12	9017095	9018122	1027	Deletion	A2ML1	Present	Present	Inherited
12	16420123	16421283	1160	Deletion	0	Present	Absent	Inherited
12	99793948	99802771	8823	Deletion	ANKS1B	Absent	Absent	De novo
12	112296544	112297279	735	Deletion	MAPKAPK5	Present	Absent	Inherited
12	122634715	122650584	15869	Deletion	0	Present	Absent	Inherited
12	127199963	127200302	339	Deletion	0	Absent	Present	Inherited
13	21951418	21951834	416	Deletion	ZDHHC20	Present	Absent	Inherited
13	81747255	81747583	328	Deletion	0	Absent	Absent	De novo
13	108560717	108561673	956	Deletion	0	Present	Absent	Inherited
14	66157661	66158245	584	Deletion	FUT8	Present	Absent	Inherited
14	73389502	73390207	705	Deletion	0	Absent	Absent	De novo
14	85139146	85139626	480	Deletion	0	Present	Absent	Inherited
14	106932640	107174930	242290	Deletion	0	Absent	Present	Inherited
15	39267016	39267370	354	Deletion	0	Present	Present	Inherited
15	93897281	93897642	361	Deletion	0	Absent	Absent	De novo
15	101051762	101052327	565	Deletion	LASS3	Present	Present	Inherited
16	11683765	11685325	1560	Deletion	0	Present	Absent	Inherited
16	25340111	25343129	3018	Deletion	0	Present	Present	Inherited
17	801626	802228	602	Deletion	NXN	Absent	Present	Inherited
17	5587702	5588656	954	Deletion	0	Absent	Absent	De novo
17	48427538	48427922	384	Deletion	LOC100288444;XYLT2	Present	Present	Inherited
18	108872	109083	211	Deletion	0	Absent	Absent	De novo
18	52447413	52447773	360	Deletion	0	Present	Absent	Inherited
18	63766868	63769201	2333	Deletion	0	Present	Present	Inherited
19	1182661	1183179	518	Deletion	0	Absent	Absent	De novo

19	12753340	12754497	1157	Deletion	0	Absent	Absent	De novo
19	14732344	14734142	1798	Deletion	EMR3	Present	Present	Inherited
20	23671899	23674222	2323	Deletion	0	Present	Absent	Inherited
20	26214307	26220376	6069	Deletion	0	Absent	Absent	De novo
20	44535505	44535941	436	Deletion	PLTP	Absent	Present	Inherited
22	35645166	35646141	975	Deletion	0	Absent	Present	Inherited
22	43101594	43102348	754	Deletion	A4GALT	Present	Absent	Inherited
22	49063815	49064594	779	Deletion	FAM19A5	Present	Present	Inherited
22	49764594	49769018	4424	Deletion	0	Absent	Absent	De novo
X	899553	899984	431	Deletion	0	Present	Absent	Inherited
X	16427688	16428223	535	Deletion	0	Absent	Present	Inherited
X	49731220	49733608	2388	Deletion	CLCN5	Absent	Absent	De novo
1	825765	5726936	4901171	Tandem Dup	ACAP3;ACTRT2;AGRN;AJAP1;ARHGEF16;ATAD3A;ATAD3B;ATAD3C;AURKAIP1;B3GALT6;C1orf159;C1orf170;C1orf174;C1orf222;C1orf70;C1orf86;C1orf93;CALML6;CCDC27;CCNL2;CDC2L1;CDC2L2;CPSF3L;DFFB;DVL1;FAM132A;FLJ14100;FLJ39609;FLJ42875;GABRD;GLTPD1;GNB1;HES4;HES5;ISG15;KIAA0495;KIAA0562;KIAA1751;KLHL17;LOC100128003;LOC100128838;LOC100129381;LOC100129534;LOC100131742;LOC100132814;LOC100133612;LOC100287685;LOC100287750;LOC100287848;LOC100287898;LOC100288202;LOC100288271;LOC100288313;LOC100288379;LOC100288479;LOC115110;LOC148413;LOC284661;LOC388588;LOC401934;LOC441869;LOC643988;LOC728661;LOC728716;LRRC47;MEGF6;MIB2;MMEL1;MMP23A;MMP23B;MORN1;MRPL20;MXRA8;NADK;NOC2L;PANK4;PEX10;PLCH2;PLEKHN1;PRDM16;PRKCZ;PUSL1;RER1;SAMD11;SCNN1D;SDF4;SKI;SLC35E2;SSU72;TAS1R3;TMEM52;TMEM88B;TNFRSF14;TNFRSF18;TNFRSF4;TP73;TPRG1L;TTLL10;UBE2J2;VWA1;WDR8	Absent	Absent	De novo
1	230107606	230107783	177	Tandem Dup	0	Absent	Absent	De novo

2	1426068	1522926	96858	Tandem Dup	TPO	Absent	Present	Inherited
2	2649502	2649583	81	Tandem Dup	0	Present	Absent	Inherited
3	75699636	75699709	73	Tandem Dup	0	Present	Absent	Inherited
4	80273622	80274729	1107	Tandem Dup	0	Absent	Absent	De novo
5	178012521	178012708	187	Tandem Dup	COL23A1	Absent	Present	Inherited
6	1053869	1054275	406	Tandem Dup	LOC285768	Absent	Absent	De novo
7	605977	606269	292	Tandem Dup	PRKAR1B	Present	Present	Inherited
7	140181586	140189220	7634	Tandem Dup	0	Present	Present	Inherited
10	125919520	125919748	228	Tandem Dup	0	Absent	Absent	De novo
10	132294621	132294747	126	Tandem Dup	0	Absent	Absent	De novo
11	131550263	131550706	443	Tandem Dup	NTM	Absent	Absent	De novo
14	24369410	24369615	205	Tandem Dup	0	Absent	Absent	De novo
14	66258294	66258410	116	Tandem Dup	0	Present	Present	Inherited
15	25468396	25470000	1604	Tandem Dup	SNORD115-29	Absent	Absent	De novo
15	63185677	63185745	68	Tandem Dup	0	Absent	Present	Inherited
19	544936	545072	136	Tandem Dup	GZMM	Absent	Absent	De novo
19	23262787	23265543	2756	Tandem Dup	0	Present	Absent	Inherited
20	24510425	24510694	269	Tandem Dup	C20orf39	Present	Absent	Inherited
2	61702932/ 61700753	61703455/ 61700765	523	Distal Dup	0	Absent	Present	Inherited
5	150319009/ 150319494	150319103/ 150319759	94	Distal Dup	LOC134466	Present	Absent	Inherited
12	12544853/ 12546495	12544955/ 12546622	102	Distal Dup	LOH12CR1	Present	Present	Inherited
12	87205701/ 87204798	87205819/ 87204806	118	Distal Dup	MGAT4C	Present	Absent	Inherited
13	21538303/ 21545834	21541177/ 21545834	2874	Distal Dup	0	Present	Absent	Inherited
16	69761819/ 69762887	69762131/ 69763048	312	Distal Dup	0	Absent	Present	Inherited

18	69711799/ 69712885	69712015/ 69712888	216	Distal Dup	0	Absent	Present	Inherited
23	2141240/ 2143040	2141701/ 2143092	461	Distal Dup	DHRSX;LOC100288477;LOC100288983	Present	Absent	Inherited
23	52886722/55678952	52892121/55679111	5399	Distal Dup	XAGE-4;XAGE3	Absent	Present	Inherited
2	133022029	17	45213172	Interchrom	CDC27	Absent	Absent	De novo

Table S2. Structural Variants unique to the affected member of Family 2 (II-2-1). Shared identity is based on a 50% reciprocal overlap rule.

Chr	Start	End	Size (bp)	SV Type	Gene(s)
1	1142719	1143140	421	Deletion	0
1	56401293	56401614	321	Deletion	0
1	62390602	62390951	349	Deletion	INADL
1	85748634	85748964	330	Deletion	0
1	91914111	91914566	455	Deletion	0
1	207292362	207293195	833	Deletion	C4BPA
1	222034158	222034490	332	Deletion	0
1	228572175	228572504	329	Deletion	0
2	8616813	8617120	307	Deletion	0
2	16271827	16274059	2232	Deletion	0
2	39071490	39071832	342	Deletion	DHX57
2	53625772	53628009	2237	Deletion	0
2	88821957	88822489	532	Deletion	0
2	194689502	194698766	9264	Deletion	0
2	206731199	206731522	323	Deletion	0
2	226039583	226040060	477	Deletion	0
3	10397475	10397834	359	Deletion	ATP2B2
3	23764073	23764403	330	Deletion	0
3	144888449	144888910	461	Deletion	0
3	185017344	185017669	325	Deletion	0
4	3612027	3612659	632	Deletion	0
4	29661122	29661450	328	Deletion	0
4	43931496	43931837	341	Deletion	0
4	44509394	44509712	318	Deletion	0

4	58087343	58087741	398	Deletion	0
4	92280245	92285214	4969	Deletion	KIAA1680
4	100023050	100023385	335	Deletion	0
4	149990582	149992581	1999	Deletion	0
4	181549440	181549754	314	Deletion	0
4	189969695	189970049	354	Deletion	0
4	190624640	190624987	347	Deletion	0
5	42165941	42167782	1841	Deletion	0
5	45004820	45014528	9708	Deletion	0
5	68724047	68724498	451	Deletion	MARVELD2
5	93916299	93916636	337	Deletion	C5orf36
5	127172332	127173282	950	Deletion	LOC728586
5	177821921	177823857	1936	Deletion	COL23A1
5	178106931	178111943	5012	Deletion	0
6	857494	857907	413	Deletion	0
6	11556106	11556426	320	Deletion	TMEM170B
6	32624895	32625860	965	Deletion	0
6	33026728	33028610	1882	Deletion	0
6	33125920	33126843	923	Deletion	0
6	50765971	50766282	311	Deletion	0
6	57297190	57301246	4056	Deletion	PRIM2
6	106983636	106983955	319	Deletion	AIM1
6	119417519	119417847	328	Deletion	FAM184A
6	133371501	133371845	344	Deletion	0
6	134062939	134063433	494	Deletion	0
6	154446930	154447253	323	Deletion	OPRM1
7	9634650	9635993	1343	Deletion	0

7	42549714	42550036	322	Deletion	0
7	158029478	158030452	974	Deletion	PTPRN2
8	98595409	98595748	339	Deletion	0
9	71895487	71896647	1160	Deletion	0
9	87775285	87776256	971	Deletion	0
9	129520461	129523267	2806	Deletion	0
9	136379222	136379564	342	Deletion	0
10	35593222	35593552	330	Deletion	CCNY
10	57702043	57702383	340	Deletion	0
10	107253002	107253355	353	Deletion	0
10	114157801	114158139	338	Deletion	ACSL5
11	7716915	7717231	316	Deletion	OVCH2
11	31916727	31917056	329	Deletion	0
11	32286653	32286986	333	Deletion	0
11	33537687	33538010	323	Deletion	0
11	65642111	65643526	1415	Deletion	0
11	104755159	104762566	7407	Deletion	CASP12
12	124458567	124496090	37523	Deletion	ZNF664
12	130060303	130060634	331	Deletion	TMEM132D
13	23055219	23055526	307	Deletion	0
13	81747255	81747583	328	Deletion	0
13	93056847	93057422	575	Deletion	GPC5
14	35428814	35432457	3643	Deletion	0
14	38643511	38643828	317	Deletion	0
14	42486335	42486650	315	Deletion	0
14	76813774	76814209	435	Deletion	0
14	78828245	78828596	351	Deletion	0

14	100845014	100845347	333	Deletion	WDR25
15	22336853	22344093	7240	Deletion	LOC727924
15	39372537	39373645	1108	Deletion	0
15	41864762	41865200	438	Deletion	TYRO3
15	42017748	42018080	332	Deletion	MGA
15	50946438	50947428	990	Deletion	TRPM7
15	67083771	67084069	298	Deletion	0
15	83773883	83774212	329	Deletion	0
15	91173990	91174326	336	Deletion	CRTC3
15	91981580	91989268	7688	Deletion	0
15	93515798	93516111	313	Deletion	CHD2
16	12002336	12002661	325	Deletion	GSPT1
16	23910887	23911193	306	Deletion	PRKCB
16	86284321	86284629	308	Deletion	0
17	136772	138726	1954	Deletion	RPH3AL
17	193726	197057	3331	Deletion	RPH3AL
17	41382745	41399871	17126	Deletion	0
17	41383489	41466010	82521	Deletion	LOC100130581
17	46904824	46907714	2890	Deletion	0
17	47228439	47228769	330	Deletion	B4GALNT2
17	57363991	57365775	1784	Deletion	0
17	69852154	69852481	327	Deletion	0
18	1199564	1200310	746	Deletion	TFG/GPR128
18	3997022	3997479	457	Deletion	0
18	22892494	22892818	324	Deletion	ZNF521
18	27629690	27630016	326	Deletion	0
18	37061646	37061962	316	Deletion	LOC647946

18	43261014	43261339	325	Deletion	SLC14A2
18	44338433	44338770	337	Deletion	0
18	50462567	50463032	465	Deletion	DCC
19	1182661	1183179	518	Deletion	0
19	2128535	2129051	516	Deletion	AP3D1
19	45473177	45474322	1145	Deletion	CLPTM1
20	25583242	25583678	436	Deletion	0
20	33241939	33244364	2425	Deletion	PIGU
20	53292665	53292978	313	Deletion	0
21	16588380	16591454	3074	Deletion	0
21	36072707	36073562	855	Deletion	CLIC6
21	39590542	39590888	346	Deletion	0
22	33295772	33296090	318	Deletion	SYN3
22	49622515	49624301	1786	Deletion	0
X	31577478	31577794	316	Deletion	DMD
X	85605177	85609869	4692	Deletion	DACH2
X	138384911	138385237	326	Deletion	0
1	30569469	30965397	395928	Tandem Dup	0
2	242929421	242929637	216	Tandem Dup	0
3	139670597	139674454	3857	Tandem Dup	CLSTN2
4	190478831	190479023	192	Tandem Dup	0
6	160877802	160956684	78882	Tandem Dup	LOC100289195;LPA;LPAL2
7	606117	606324	207	Tandem Dup	PRKAR1B
7	156124573	156124795	222	Tandem Dup	0
9	44070664	44070787	123	Tandem Dup	LOC100289454
9	140563766	140564031	265	Tandem Dup	0
10	27224456	27229304	4848	Tandem Dup	C10orf51

13	34811550	34815591	4041	Tandem Dup	0
14	105943990	105944114	124	Tandem Dup	CRIP2
19	14706528	14706835	307	Tandem Dup	CLEC17A
X	52886720	55678950	2792230	Tandem Dup	XAGE4;ALAS2;APEX2;FAM104B;FAM120C;FAM156A;FAM156B;FGD1;FOXR2;GNL3L;GPR173;HSD17B10;HUWE1;IQSEC2;ITIH5L;KDM5C;LOC100132984;LOC100288024;LOC100288052;LOC100288498;LOC100288560;LOC644893;MAGED2;MAGEH1;PAGE2;PAGE2B;PAGE3;PAGE5;PFKFB1;PHF8;RIBC1;SMC1A;SNORA11;TRO;TSPYL2;TSR2;USP51;WNK3;XAGE3
2	16407764/ 16406392	16407988/16406410	224	Distal Dup	0
3	194546260/ 194543294	194546429/194543309	169	Distal Dup	0
7	100550585/ 100551244	100550785/100551247	200	Distal Dup	MUC3A
9	107816635/ 107817341	107816979/107817348	344	Distal Dup	0
23	17060848/ 17063276	17061023/17063295	175	Distal Dup	REPS2
